# Ginsenoside Compound K: Insights into Recent Studies on Pharmacokinetics and Health-Promoting Activities

**DOI:** 10.3390/biom10071028

**Published:** 2020-07-10

**Authors:** Anshul Sharma, Hae-Jeung Lee

**Affiliations:** 1 Department of Food and Nutrition, College of Bionanotechnology, Gachon University, Gyeonggi-do 13120, Korea; anshul.silb18@gmail.com; 2 Institute for Aging and Clinical Nutrition Research, Gachon University, Gyeonggi-do 13120, Korea

**Keywords:** ginseng, compound M1, hepatoprotective, anti-cancer, anti-inflammation, anti-diabetic, safety

## Abstract

Ginseng (*Panax ginseng*) is an herb popular for its medicinal and health properties. Compound K (CK) is a secondary ginsenoside biotransformed from major ginsenosides. Compound K is more bioavailable and soluble than its parent ginsenosides and hence of immense importance. The review summarizes health-promoting in vitro and in vivo studies of CK between 2015 and 2020, including hepatoprotective, anti-inflammatory, anti-atherosclerosis, anti-diabetic, anti-cancer, neuroprotective, anti-aging/skin protective, and others. Clinical trial data are minimal and are primarily based on CK-rich fermented ginseng. Besides, numerous preclinical and clinical studies indicating the pharmacokinetic behavior of CK, its parent compound (Rb1), and processed ginseng extracts are also summarized. With the limited evidence available from animal and clinical studies, it can be stated that CK is safe and well-tolerated. However, lower water solubility, membrane permeability, and efflux significantly diminish the efficacy of CK and restrict its clinical application. We found that the use of nanocarriers and cyclodextrin for CK delivery could overcome these limitations as well as improve the health benefits associated with them. However, these derivatives have not been clinically evaluated, thus requiring a safety assessment for human therapy application. Future studies should be aimed at investigating clinical evidence of CK.

## 1. Introduction

Ginseng (*Panax ginseng*) of the Araliaceae family is a perennial plant which has been conventionally used as a functional food. It is commonly consumed as a health supplement in Korea, Japan, China, the United Kingdom, Canada, Germany, France, and Austria [1]. Ginseng’s bioactive constituents, including ginsenosides, phenolic compounds, and polysaccharides, have several medical uses [2]. Ginsenosides (or panaxosides) are the key pharmacologically significant bioactive constituents of ginseng. Nearly 150 ginsenosides isolated from roots, fruits, leaves, flower buds, processed items of ginseng, and other species have been identified [3]. A category of triterpene and saponin ginsenosides are divided into two forms: tetracyclic triterpenoids (four-ring dammarane type) and pentacyclic triterpenoids (five-ring oleanolic type) [4]. The dammarane type saponins are further categorized into protopanaxadiol (PPD) and protopanaxatriol (PPT) saponins [5]. The ginsenosides of PPD group constitute Compound K (CK), Rg3, Ra1, Ra3, Ra2, Rh2, Rb1, Rb3, Rb2, F2, Rc, and Rd, while PPT group constitutes of F1, Rg1, Rg2, Rf, Re, and Rh1 [3,6]. The oleanolic type saponins, such as Ro, are very low in concentration and thus rarely detected [4,6]. Examples of rare ginsenosides are CK, Rg3, F2, and Rh2, which are either absent in unprocessed ginseng or available at low concentrations [7]. It is well-known that compared to ginsenosides, their metabolite CK is absorbed well by the body and in lieu of this property, CK is becoming the fast focus of research [8]. 

**Figure 1:**
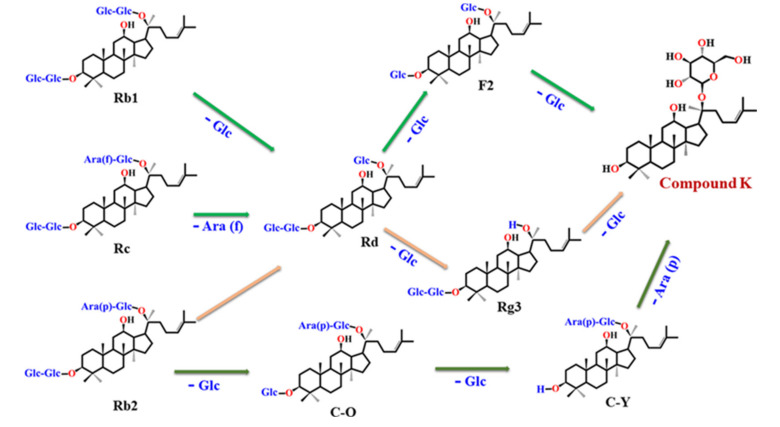
Schematic illustration of plausible biotransformation of major ginsenosides Rb1, Rb2 and Rc to compound K.

Ginsenoside CK (G-CK; 20-*O*-β-D-glucopyranosyl-20(*S*)-protopanaxadiol) is a minor tetracyclic triterpenoid, also known as IH901, CK, and M1 [9]. Compound K is absent from natural ginseng and was isolated by researchers from Japan in 1972 by several biotransformation approaches from saponins such as Rc, Rb1, and Rb2 [10]. The various processes used for the CK synthesis include enzymatic use [11], microbial conversion [3,4,12], heating [13], mycelial fermentation [14] and metabolic engineering [15]. The detailed procedure of biotransformation has been described in detail elsewhere [3]. Compound K is a major deglycosylated metabolite found in organs or blood after oral ingestion of PPD ginsenosides in the human gastrointestinal (GI) tract (Figure 1) [11]. The molecular weight and chemical formula of CK are 622.86 g/mol and C_36_H_62_O_8_, respectively [16]. 

A review by Yang et al. [11] described in detail the biotransformation of CK and recorded its pharmacological activities until 2014. Another review [3] compiled information on the biotransformation and pharmacokinetics of CK, including the positive effects on neuroprotection and cognitive improvement by 2016. Like the previously published reviews, this paper documents recent papers targeting hepatoprotective, anti-inflammatory, anti-atherosclerosis, anti-diabetic, anti-cancer, neuroprotective, anti-aging/skin protective effects published from 2015 to 2020. This review, however, differs significantly from previous works, including detailed information on preclinical and clinical pharmacokinetic studies and the inclusion of anti-asthmatic and kidney-protective effects among others in vitro and in vivo activities of CK. Additionally, data are also integrated on several CK derivatives with improved solubility and health-promoting activities. Finally, recently published clinical studies are also summarized. Herein, we briefly discuss the recent reports on the pharmacokinetics and health effects of CK and elucidate on how the modification of CK improves metabolic properties, pharmacokinetics, and bioactivities of CK.

## 2. Literature Search 

A precise literature search was carried out with Google Scholar, PubMed, and the Science Direct repositories for related findings between January 2015 and May 2020. The following keywords: “Ginsenoside compound K” or “Compound K” or “20-*O*-D-glucopyranosyl-20(*S*)-protopanaxadiol” or “IH901” or “M1” or “health-promoting activities of compound K,” or “Ginsenoside Compound K and pharmacokinetics,” “Compound M1,” and “fermented ginseng,” were used to find all the relevant literature published on CK, its pharmacological activities, and modifications conferred on its structure so as to augment solubility and bio permeability. 

## 3. Pharmacokinetics, Metabolism and Safety of Compound K

Compound K has recently been the focus of research for its bioactivities; however, little is known about its pharmacokinetic behavior, solubility, bioavailability and safety. 

### 3.1. Preclinical Perspective (Pharmacokinetic)

It is important to have an in-depth knowledge of how pro-and prebiotics affect adsorption, distribution, metabolism, and excretion (ADME) of drugs. In this line, the effect of prebiotic fiber (NUTRIOSE^®^) on the pharmacokinetic behavior of CK was investigated using Sprague-Dawley (SD) rats. NUTRIOSE^®^ treatment displayed a dose-dependent rise in plasma concentration (C_max_), area under the plasma concentration-time curve (AUC), and a decrease in time of maximum concentration (T_max_) values compared to control. Moreover, the increased glycosidase activity led to the synthesis of CK in the internal intestinal constituents. In addition, NUTRIOSE^®^ has substantially triggered the biotransformation of CK by in vitro grown gut microbiota [17]. This study was in par with earlier findings wherein gut microbiome and diet were reported for possible effects on the concentration of CK in plasma [8,18]. 

In a study, fermented and non-fermented red ginseng extracts were administered to SD rats. Based on the pharmacokinetics parameters, CK absorption was found to be more in the fermented group than non-fermented group (Table 1) [19]. In another study, the pharmacokinetics of the oral administration of *P. ginseng* extract was evaluated in plasma, urine, and fecal samples using SD rats. The study used liquid chromatography (LC)-mass spectrometry (MS)/MS for identifying and quantifying major saponins and their metabolites present in *P. ginseng* extract. The C_max_, T_max_, and AUC, were significantly higher for CK (in rat plasma) compared to other ginsenosides, including Re, Rg1, Rc, Rf, Rb1, Rg2, Rb2, Rd, F1, and F2. The study for the first time documented the comprehensive pharmacokinetics of ginsenosides and their metabolites following oral intake of *P. ginseng.* Earlier studies documented the pharmacokinetic properties of several individual ginsenosides only [20]. 

In another study from Japan, the oral pharmacokinetic behavior of CK/gamma-cyclodextrin (K/γ-CyD, 1:1) in SD rats has been described. The strong dissolution behavior of CK (K/γ-CyD) was attributed to a higher significant change in C_max_, AUC values and a lower T_max_ compared with CK and K/β-CyD (Table 1). The stability constant for (K/γ-CyD, 1:1) complex was 18 times (1.5 × 10^5^ M^−1^) than that of the β-CyD complex (8.2 × 10^3^ M^−1^). The compound-complex (K/γ-CyD, 1:1) displayed a faster dissolution rate compared to K/γ-CyD complex at 1:3 ratios. The study concluded that partial inclusion complexes are more advantageous for improving the solubility of CK compared to complete inclusion [21]. 

### 3.2. Clinical Perspective (Pharmacokinetic)

Previously, the pharmacokinetics of CK was studied as part of the ingestion of whole ginseng extract. In one study, the intake of fermented red ginseng extract showed a higher concentration (more than 10 times) of CK in plasma compared to its unfermented counterpart in a study of healthy Korean volunteers. In the fermented group, AUC was 15.5-fold higher than the non-fermented group, and the mean C_max_ was 27-fold higher compared to the unfermented group. A lower T_max_ was observed in the fermented group than non-fermented group (Table 1). Compared to a previous study [22] where subjects were administered with 5 g of Korean red ginseng, this study showed larger AUC, higher C_max_, and longer T_max_ values with lower (3 g) amount of fermented red ginseng. The authors described the differences in the pharmacokinetic parameters plausibly due to different methods used for fermentation of red ginseng. Of note, the study also confirmed these effects in vivo and found absorption of CK was more in humans compared to rats. The study also highlighted that absorption of CK depends upon the interspecies variability between humans and rats [19]. It has also been reported that the fermentation of white ginseng had less effect on CK absorption compared with red ginseng fermentation [23].

Likewise, another study compared the pharmacokinetics of CK after oral administering fermented ginseng (FG, by *Lactobacillus (L.) paracasei* A221) and non-fermented ginseng (NFG) in healthy Japanese adults. A higher C_max_ and a lower T_max_ value of CK was observed for FG compared to NFG (Table 1). The mean AUC value from 0 to 12 h was 58.3-times, and AUC value from 0 to 24 h was 17.5-times higher than the NFG. Furthermore, following 24 h supplementation, the mean concentration of testosterone was increased significantly in the male subjects treated with FG [24]. The findings concluded that the transformation of ginseng extract by *L. paracasei* A221 resulted in improved health in Japanese subjects. This is the first research on Japanese subjects which evaluates the health properties of *P. ginseng*. The positive bio transforming role of *L. paracasei* A221 modulating bioavailability and functional aspects has also been described [25]. In recent research, LC-MS was used to spot 13 ginsenosides in the human plasma following two-week recurrent supplementation of red ginseng extract. Among 13 ginsenosides, CK, Rh2, PPD, and PPT were detected in the subject’s plasma, although initially not presented in red ginseng extract, suggesting the formation of these ginsenosides after bioconversion in the GI tract. The C_max_, T_max_, and AUC values were found to be higher for CK among 13 ginsenosides. The authors further identified a large variation in the concentration of CK among subjects owing to their metabolic differences associated with microorganisms of the GI tract (Table 1) [26]. 

In a recent randomized, open-label, single-sequence study, the participants were administered with the red ginseng product of a single dose and two-week repeated dose. The quantities of CK, Rc, Rb1, Rd, and Rb2 ginsenosides in plasma of the human subjects were measured. Of the 15 participants, three subjects revealed higher plasma levels of CK and Rd, suggesting higher bioconversion of Rb1, Rb2, and Rc to Rd and then to CK. The study showed that the multiple-dose of red ginseng extract did not boost the AUC and C_max_ values, leading to low accumulation of CK (compared to other ginsenosides) in plasma, due to the comparatively short half-life of CK (Table 1). Furthermore, the AUC values of CK and Rd were significantly correlated irrespective of the dose amount. These results suggest the upstream biotransformation of saponins and have found that the repeated dose of CK is healthy for human consumption [27]. 

Apart from ginseng extracts or a mixture of ginsenosides, the pharmacokinetics of monomer CK has also been described [28,29]. In one study, LC-MS/MS (positive ion mode) with lithium adducts, was used to measure CK concentration in plasma of healthy Chinese subjects. Adducts were intended to boost the MS functionality. The method used was found to be perfect, reproducible and precise compared to the LC-MS/MS (negative ion mode) study reported earlier [22] for CK determination in human plasma. In addition, the lower limit of quantification was achieved by using smaller concentrations (50 μL) compared to higher human plasma levels (100 μL) [29]. An open-label, single-center, randomized, two-period crossover trial found that high-fat food consumption with CK decreased its T_max_ value and increased its values for C_max_ and AUC relative to the overnight fasting community (Table 1). Moreover, in females, the CK consumption was higher than in males. These results suggest that both food (high-fat diet) and sex affect the pharmacokinetics of CK and its metabolite, 20(*S*)-PPD. Such findings revealed preliminary pharmacokinetics of pure CK and its metabolite. To make a stronger conclusion, further research through large population size and daily diet record should be implemented [28]. Another recent, randomized, single-center, open-label, two-period crossover trial study first applied and validated LC-MS/MS to govern the pharmacokinetics of CK and its 20 (*S*)-PPD metabolites present in plasma and urine samples of healthy Chinese volunteers (Table 1) [30].

**Table 1:** Pharmacokinetics of CK and its derivatives.

Subject	Compound	Dose			Pharmacokinetics Parameters					Ref.
			C_max_ (ng/mL)	T_max_ (h)	AUC (ng·h/mL)	V/F (L)	MRT (h)	CL/F (L·h^−1^)	T_1/2_ (h)	
**Preclinical Studies**										
SD rats	GE (N0-G)	2000 mg/kg	24.1 ± 5.5	15.2 ± 1.8	153.1 ± 30.6					[17]
	GE ± 2.5% N		24.0 ± 9.3	12.8 ± 3.3	187.2 ± 24.0					
	GE ± 5% N		38.8 ± 21.8	12.0 ± 0.0	218.5 ± 60.7					
	GE ± 10% N		54.4 ± 26.2	12.0 ± 0.0	429.9 ± 160.8					
SD rats	HYFRG™ ***	500 mg/kg	15.19 ± 10.69	3.3 ± 0.5	58.0 ± 32.5					[19]
	CK from RG	500 mg/kg	2.55 ± 0.99	6.7 ± 3.9	9.2 ± 7.5					
SD rats	CK from PG ^#^	100 mg/kg	1888.9 ± 403.0	8.2 ± 1.7	24.0 ± 6.0		13.9 ± 5.2		10.2 ± 8.1	[20]
SD rats	CK	30 mg/kg	192.3 ± 40.7	2.2 ± 0.5	622.3 ± 240.7		3.8 ± 0.8			[21]
	CK/γ-CyD (1:1)		366.7 ± 102.5	1.8 ± 0.1	907.3 ± 111.1		2.5 ± 0.2			
	CK/γ-CyD (1:3)		476.0 ± 81.5	1.5 ± 0.2	1074.8 ± 32.9		2.2 ± 0.0			
	CK/β-CyD (1:1)		204.0 ± 30.8	2.0 ± 0.4	867.0 ± 69.6		3.3 ± 0.5			
**Clinical Studies**										
24 M	HYFRG™	3 g	254.4 ± 51.2	2.5 ± 0.9	1466.83 ± 295.89					[19]
	RG	3 g	3.1 ± 1.7	9.1 ± 1.4	12.73 ± 7.83					
12 M/F	FG	1.65 g	41.5 ± 21.8	2.2 ± 0.6	204 ± 94 (0–12 h), 238 ± 105 (0–24 h), 264 ± 113 ^$$^		9.9 ± 5.5		10 ± 5	[24]
	NFG		1.1 ± 0.7	16 ± 7.0	3.5 ± 3.1 (0–12 h), 13.6 ± 9.3 (0–24 h), NC		NC		NC	
11 M	RG extract ^##^	Multiple	81.6 ± 112.5	9.5 ± 1.6	873.0 ± 1236.0		10.6 ± 1.2		5.2 ± 1.1	[26]
15 M	RG extract ^###^	Single	24.8 ± 23.2	7.8 ± 2.0	247.50 ± 269.49		13.3 ± 3.7		9.9 ± 4.9	[27]
		Multiple	18.2 ± 27.1	6.9 ± 2.4	210.88 ± 400.44		10.5 ± 3.1		7.6 ± 4.1	
24 M/F	CK + HF diet	200 mg	1,570.3 ± 587.3	2.5 (1.5–5.0)	12,599.2 ± 4098.3 ^$^; 12,836.7 ± 4166.2 ^$$^	652 ± 381	12.3 ± 1.2^$^, 14.6 ± 1.7 ^$$^	18.2 ± 9.8	24.8 ± 3.0	[28]
	CK + FO diet		796.8 ± 406.0	3.6 (2.0–6.0)	5748.7 ± 2830.2 ^$^; 5879.3 ± 2871.0 ^$$^	1875 ± 1899	11.7 ± 1.2 ^$^, 15.1 ± 4.3 ^$$^	43.4 ± 24.2	27.7 ± 7.9	
12 M/F	GCK	50 mg	652 ± 180	2.6 ± 1.1	3650 ± 850 ^$^; 3810 ± 890 ^$$^				5.9 ± 0.6	[29]
10 adults	CK	200 mg	733.9 ± 408.4	3.3 (2.5–5.0)	5960.8 ± 3524.4 ^$^; 6094.2 ± 3598.4 ^$$^		11.5 ± 1.4 ^$^, 13.8 ±1.6 ^$$^		21.6 ± 5.5	[30]

*** The study was conducted on both rats and humans. ^#^ analysis was compared with other ginsenosides, including Rb1, Rb2, Rc, Rd, Re, Rg1, Rg2, Rf, F1 and F2. ^##^ RG extract dose protopanaxadiol (PPD)-50.2–64.7 mg/day and protopanaxatriol (PPT), 11.2–14.9 mg/day. Multiple (3 Pouches). CK was compared with Rb1, Rb2, Rc, Rd, Rg3, Rh2, PPD, and PPT. ^###^ RG extract consisted of a pouch with > 60% of dried ginseng extract. Multiple (3 Pouches). CK was compared with Rb1, Rb2, Rc and Rd. ^$^ AUC _(0-th)_, ^$$^ AUC _(0-∞ h)_. CK, compound K; C_max_, maximum drug concentration; T_max_, time of maximum concentration; AUC, area under the plasma concentration-time curve; V/F, apparent volume of distribution after extravascular administration MRT, mean residence time; CL/F, oral clearance; T_1/2_, half-life. Each value represents the mean ± SE of three samples. SD, Sprague Dawley; GE, ginseng extract; N, NUTRIOSE; HYFRG™, CK from fermented RG extract; RG, red ginseng; PG, *Panax ginseng*; M., Male; F, female; FG, fermented ginseng (fermented using *Lactobacillus paracasei* A221); NFG, non-fermented ginseng; NC, not-calculated; GCK, ginsenoside CK; AUC _(0-th)_, AUC from zero to the time of the last quantifiable concentration; AUC_(0-∞h)_, AUC from zero to infinity; HF, high fat; FO, fasting overnight.

Furthermore, a study on healthy Chinese participants has established the relationship between ABCB1 gene polymorphisms and CK pharmacokinetics. The results indicated that the gene NR1I2 (rs1464602 and rs2472682) allied primarily to the pharmacokinetics of CK. While ABCC4 (rs1751034 and rs1189437) influenced the pharmacokinetic behavior of both CK and its metabolite 20(*S*)-PPD. Such hereditary variations could thus partially describe the inter-individual variances in the pharmacokinetic behavior of CK [31]. 

### 3.3. Solubility, Permeability, and Efflux

Many health-promoting activities of CK have been reported. However, low water solubility, low membrane permeability, and efflux phenomenon critically weaken its efficacy and restrict its clinical application. The use of cyclodextrin (CyD) and nanocarriers have been implemented to improve the bioavailability of CK. Table 2 summarizes various modifications of CK with their outcomes. 

The use of CyD has been duly recognized to improve the pharmacological behavior of drugs. In this line, an inclusion complex, K/γ-CyD, with improved oral bioavailability and solubility [21], compared to an earlier finding (in β-CyD only solubility was improved) has been described [32]. In another study, the use of ginsenoside CK with TPGS (d-alpha-tocopheryl polyethylene glycol (PEG) 1000 succinate) (GCKT)-liposomes has been described to improve solubility, targeting tumor cells, and minimizing efflux. The d-alpha-tocopheryl polyethylene glycol (PEG) 1000 succinate and phospholipid could increase the solubility of CK in the form of GCKT-liposome, leading to significant repression of tumor growth [33]. Phospholipid use improves biocompatibility, which could restore permeability and increase the process of ADME [34]. The d-alpha-tocopheryl polyethylene glycol (PEG) 1000 succinate has widely been documented as an inhibitor of P-glycoprotein (P-gp)-mediated efflux in drug delivery systems [35], and P-gp-mediated efflux was reported to be a significant limiting factor for the efficacy of CK [36]. 

In another study, CK-micelles (CK-M) from TPGS, PEG, and PCL (polycaprolactone) showed enhanced solubility and improved bioactivities. After 48 h, the CK was released slowly from CK-M with a drug release percentage of more than 42.1 ± 3.2% and without bursting. In the first eight hours, the rate of in vitro drug release for free CK with bursting was 84.4 ± 4.2%. Additionally, the P-gp-mediated efflux in the CK-M group was substantially inhibited compared to free CK, suggesting drug uptake by the target cells [36]. Likewise, CK ascorbyl palmitate (AP)/TPGS micelles enhanced solubility of CK and significantly inhibited P-gp-mediated efflux [37]. Similarly, the micellar system based on phosphatidylcholine (PC) and 1,2-distearoyl-sn-glycero-3-phosphoethanolamine polyethylene glycol 2000 (DP) showed improved solubility and continued release of CK [38]. The water solubility of the CK nanoparticles (NPs)/bovine serum albumin (BSA) and CK, was compared, and BSA was found to augment the water solubility of CK. The high biocompatibility, dispersive nature, and conjugative ability to several target molecules make BSA a useful carrier molecule [39]. 

In another study, CK was loaded onto gold(G)NPs synthesized using probiotic bacteria (*Lactobacillus kimchicus* DCY51^T^) and evaluated for the effectiveness of drug loading [40]. Furthermore, the use of deoxycholic acid (DA)-*O*-carboxymethyl chitosan (OCMC) has been advocated to increase solubility and ability for CK. For example, CK-NPs conjugated with DA-OCMC showed increased solubility and improved drug entrapping and drug loading efficiencies. The release pattern of CK was pH-dependent and faster at lower pH. The collective release of CK at pH 7.4 and 5.8 was 10.7 ± 0.71%, and 16.3 ± 1.4%, respectively, after the first 48 h, without bursting. Notably, over 120 h of the study, a significant increase in the release of CK was observed. These findings indicate that CK was released slowly (the pH of blood), thus the system could be used for target delivery of CK [41].

**Table 2:** Solubility, permeability, and efflux of CK and its derivatives.

Modified CK	Model	Major Findings	Ref.
K/γ-CyD and K/β-CyD	K/γ-CyD at different ratios 1:1 and 1:3 and K/β-CyD at 1:1	Improved solubility at lower concentrations (<0.03 M) compared to higher (<0.06 M) ↑bioavailability ↑dissolution rate compared to CK and K/β-CyD Higher dissolution rate in 1:1 ratio compared to 1:3	[21]
GCKT-liposomes	Phospholipid and TPGS (7:3 ratio)	↑High CK loading capacity and solubility GCK EE% was of above 98.4 ± 2.3% Sustained discharge of GCK from GCKT-liposomes compared to GCK solution (in PBS)	[33]
CK-M	PEG-PCL/TPGS mixed micelles at different ratios of 3:0. 3:1, 3:2, 3:3	↑drug EE% in CK-M (94.6 ± 1.4) than CK-P (62.5 ± 1.6; PEG-PCL micelles) ↑CK concentration (107.3-times) in micelles (CK-M) than free CK ↑solubility of CK with higher TPGS	[36]
CK-AP/TPGS micelles	AP/TPGS mixed micelles	↑solubility from 35.2 ± 4.3 to 1,463.2 ± 153.3 µg/mL of CK EE% = 91.3 ± 5.2% Inhibited P-gp mediated efflux	[37]
CK PC/DP micellar system	CK, DP, and PC at ratios of 5:12:18	↑water solubility (~66-fold) and long drug retention time	[38]
BSA-CK NPs	BSA	↑water solubility	[39]
DCY51T-AuCKNps	AuNPs synthesized using *Lactobacillus kimchicus*	Drug loading efficiency-11.03%	[40]
CK:DA-OCMC NPs	CK:DA-OCMC at different ratios 1:10, 2:10, 3:10	↑water solubility ↑EE% from 20.2 ± 1.4 to 42.6 ± 1.2% ↑drug loading capacity from 3.0 ± 0.2 to 10.6 ± 1.4% by ↑drug: carrier ratio Enhanced cellular uptake and increased cytotoxicity than CK	[41]
GK-OCMC NPs	GK: OCMC at different ratios of 1:10, 2:10, 3:10	↑water solubility and permeability ↑EE% from 5.9 ± 1.2 to 20.8 ± 2.5% ↑drug loading capacity from 1.9 ± 1.8 to 4.2 ± 0.7 % by ↑ drug: carrier ratio Enhanced cellular uptake and increased cytotoxicity than CK	[42]
APD-CK micelles	CK: A54-PEG-DA-OCMC at different ratios of 1: 20, 2:20, 4:20	↑EE% increased from 61.7 ± 1.4 to 76.5 ± 1.2 % ↑drug loading capacity from 1.6 ± 0.1 to 3.1 ± 1.4 % by ↑drug: carrier ratio	[43]

CK, compound K; CyD, cyclodextrin; GCKT, ginsenoside CK with TPGS; TPGS, D-α-tocopheryl polyethylene glycol 1000 succinate; EE, encapsulation efficiency; PEG, polyethylene glycol; PCL, polycaprolactone; AP, ascorbyl palmitate; P-gp, P-glycoprotein; PC, phosphatidylcholine; DP, 1,2-distearoyl-sn-glycero-3-phosphoethanolamine polyethylene glycol 2000; BSA, bovine serum albumin; NPs, nanoparticles; OCMC, O-carboxymethyl chitosan; DA, deoxycholic acid.

In a recent comparative study on CK and CK within OCMC NPs showed that the later had higher water solubility and membrane permeability [42]. Similarly, a recent study found that CK-loaded with A54-PEG-DA-OCMC, known as APD-CK micelles, enhanced the delivery of CK. A54 is a long peptide of 12 amino acids which binds explicitly to hepatic cancer cells. Drug release was pH-dependent, and its release at pH 7.4 was slow (32.69%) compared to a fast release (73.49%) at pH 5. 8 [43].

### 3.4. Safety

As per the new clinical guidelines, drug safety tests should be screened with two animal types, inclusive of non-rodents (usually dogs) and rodents (mice or rats) [44]. For preclinical safety evaluation, rats and mice were assessed for acute and 26-week recurrent-dose toxicity of CK.

Single oral supplementation of CK for rats (8 g/kg) and mice (10 g/kg) did not induce toxicity or mortality in the same. On the other hand, for 26-week toxicity (e.g., clinical symptoms, biochemical and hematological parameters, urine analysis, the body weights, food consumption, and histopathology of rats) were evaluated at 13, 40, or 120 mg/kg doses of CK. The NOAEL (no observed adverse effect levels) doses were 40 mg/kg and 120 mg/kg for females and males, respectively. However, a decrease in body weight, fur-loss, reduced activity, and lack of energy were transiently observed in the 120 mg/kg male test group [45]. Oral preclinical safety of CK was evaluated on Beagle dogs (4, 12, or 36 mg/kg) for 26-weeks. The NOAEL dose for dogs was 12 mg/kg [46]. 

Considering clinical perspective, in a randomized, double-blind trial on healthy Chinese subjects, compared to placebo, the treatment group were orally administered CK at 100, 200, or 400 mg doses for up to nine times a week. The results of this documented study showed the safety of CK during the intervention period [47]. However, it has been suggested that further evaluations are necessary to affirm the safety of CK administration in humans. 

## 4. Health-Promoting Activities

**Figure 2:**
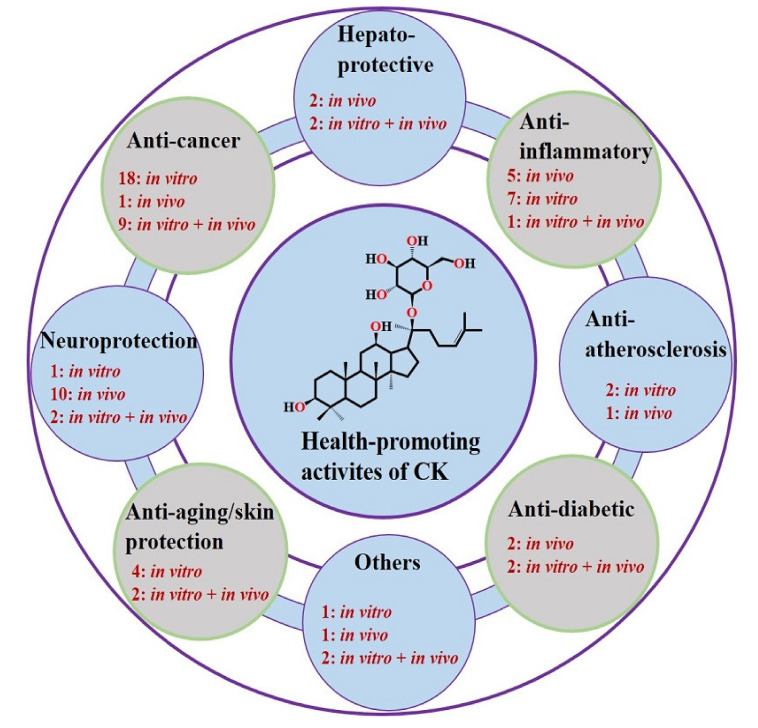
Schematic illustration of health-promoting activities of Compound K (CK). The numbers in the figure represent the total number of studies assessed for evaluating in vitro and in vivo, health-promoting activities of CK.

Compound K, in terms of its bioactivity, has gained much interest as a rarely known ginsenoside [48]. Investigation on the CK metabolism is beneficial to gain better insights into the pharmacological activities of CK. Concerning this, a recent study used an ultra-performance LC quadrupole time-of-flight tandem MS to characterize CK (oral dose 50 mg/kg) in feces and urine of SD rats, resulting in the detection of tentative twelve (M1–M12) metabolites. The authors suggested sequential oxidation, deglycosylation and conjugation as the key metabolic pathways for CK metabolic profile characterization [49]. This section summarizes recent studies on various health-promoting activities of CK (Table 3 and Table 4 and Figure 2). 

### 4.1. Hepatoprotective

The hepatoprotective effects of CK were observed against the injury caused by carbon tetrachloride [50], tert-butyl hydroperoxide [51], paracetamol (acetaminophen) [52], or as in recent studies by sodium valproate (SVP) [9]. Compound K exhibited protective effects against hepatotoxicity caused by SVP via minimizing oxidative stress through triggering the hepatic antioxidant system and inhibiting lipid peroxidation [9]. Among other protecting effects, CK significantly improved liver fibrosis in a high-fat diet (HFD)-induced rats [53]. Another study evaluated the effect of CK on hepatosteatosis using a mouse model with diabetes and obesity. The beneficial effects against hepatosteatosis were described by reducing expressions of lipogenesis genes and upregulating expressions of genes involved in fatty acid oxidation through adenosine monophosphate-activated protein kinase (AMPK) phosphorylation [54].

**Table 3:** Hepatoprotective, anti-inflammatory, anti-atherosclerosis, and anti-diabetic activities of CK and its derivatives.

Material Type	ST	Model	Treatments	Major Findings	Ref.
**Hepatoprotective**					
CK	In vivo	SVP-induced SD rats	LCK-80 mg/kg GCK + SVP MCK-160 mg/kg GCK + SVP HCK-320 mg/kg GCK + SVP once daily for 15 days	↓ hepatic index-LCK (7.6%), MCK (8.7%), and HCK (9.4%) ↓ AST, ALT, ALP, TG and ↑ALB ↑ CAT, GPx, and SOD activities and GSH level ↓ MDA level and soluble epoxide hydrolase (better with LCK) ↑ hepcidin level	[9]
CK and Rh1	In vivo	HFD-treated SD rats	CK + phospholipid; phospholipid + Rh1; phospholipid + CK+ Rh1 (3 mg/kg/day), 1 week	Treatment either alone or in combined form (CK or Rh1) ↓ γ-GT, AST, ALT, ALP, TG, CHOL, FCHOL, LDL ↑ HDL levels Anti-fibrotic effects by ↓ expressions of TIMP-1, PC-I, and PC-III Improved insulin resistance by normalizing glucose levels	[53]
	In vitro	Rat liver stellate cell line (HSC-T6)	CK, Rh1, CK+Rh1 for 6 h	↑anti-proliferative effect ↑ apoptosis in HSC-T6 CK (20.63%), Rh1(12.43%), CK+Rh1 (18%)	
CK	In vivo	HFD-treated OLETF rats	CK (25 and 10 mg/kg), 12 weeks	↓ plasma glucose level and improved morphology of liver cells ↓ FAS and SREBP-1c expressions ↑ CPT-1 and PPAR-α expressions ↑ phosphorylation of AMPK	[54]
CK from GBCK25	In vivo	C57BL/6 mice	GBCK 25 with CK (400, 200, 100, 20, and 10 mg/kg) once daily, 12 weeks	↓ liver weight ↓inflammation, degree of steatosis, and ballooning degeneration ↓ ALT, TC and TG levels ↓ TNF-α, IL-1β, IL-6 levels ↓ expressions of α-SMA and TIMP-1 Reduction in hepatic lipid accumulation and ↓ MDA levels ↓ FAS, ACCα and CYP2E1 levels ↓ levels of p-JNK (reduced JNK activation)	[55]
	In vitro	Palmitic acid-treated AML12 cells LPS-treated RAW264.7 cells Kupffer cells (KCs)*	GBCK25 (4, 2, and 1 μg/mL), 24 h GBCK25 (0.5, 0.4, or 0.3 μg/mL), 24 h	↓ cellular toxicity ↓ TG, FAS, ACCα and CYP2E1 levels ↓ TNF-α, IL-1β, IL-6 in RAW264.7 and KC cells	
**Anti-inflammatory**					
CK	In vitro	LPS-stimulated RAW264.7 cells and HEK293 cells transfected with HA-AKT1, HA-Src, or HA-AKT2 for 48 h	CK (10, 5, and 2.5 μM), 24 h	No effect on the viability ↓ expressions of TNF-α, IL-1β, iNOS, and AOX1 ↓ phosphorylation of Akt1, not Akt2	[2]
BIOGF1K	In vitro	Pretreated RAW264.7 cells	BIOGF1K (200, 100, and 50 μg/mL), 1 h + LPS (1 μg/mL), 24 h	↓ NO production (67%) with BIOGF1K (200 μg/ mL) Significant scavenging of DPPH ↓ expressions of iNOS and IFN-β ↓ NF-kB activity (72%), IRF3 pathway (63%) Inhibited IKK and TBK1 phosphorylation	[56]
BIOGF1K	In vitro	Pretreated RAW264.7 cells	BIOGF1K (30, 20, and 10 μg/mL), 30 min + LPS (1 μg/mL), 24 h	Dose-dependent ↓ of NO and iNOS and COX-2 expressions AP-1 signaling pathway inhibited by blocking MAPKs and MAPKKs	[57]
BSA-CK NPs	In vitro	Pretreated RAW 264.7 cells	BSA-CK NPs and CK (20,15, 10, 5, and 1 µM), 1 h + LPS (1 mg/mL)	↓ NO production by BSA-CK NPs (10 µM) compared with CK	[39]
SPIONs-CK	In vitro	Pretreated RAW 264.7 cells	SPIONs-CK and CK (100, 10, and 1 μg/mL), 24 h + LPS (1 μg/mL) Antioxidant-1 to 250 μg/mL	↓ NO production by CK and SPION-CK and inhibited iNOS production by 47.9% (CK) and 45.8% (SPION-CK) (at 10 μg/mL) ↓ ROS production by SPIONs-CK and CK Inhibition of DPPH was higher for SPIONs-CK (72%) compared to CK (21.1%) at (250 μg/mL)	[58]
CK	In vivo	C57BL/6 mice	CK (20 mg/kg), 30 h	↑expression of SGLT1 gene and glucose uptake mediated by SGLT1	[59]
	In vitro	Caco-2 cells	CK (1, 0.1, 0.01, and 0.001 µM), 12, 24, 36, and 48 h	↑ SGLT1 protein level dose-dependent ↑ SGLT1 protein level time-dependent 1.70 times (24 h) to 2.01 times (48 h) ↑ glucose uptake activity by ↑ SGLT1 expressions	
CK	In vivo	Xylene-induced Kunming mice with ear swelling	CK (224, 112, 56, 28, 14, and 7 mg/kg) every day, 5 days	CK displayed a dose-dependent inhibitory effect At 224 mg/kg- maximum (93.9%) inhibition	[60]
		Carrageenan-induced paw oedema SD rats	CK (160, 80, 40, 20, 10, and 5 mg/kg), orally every day, 5 days	Pain threshold induced by heat not effected ↑ rat inflammatory pain threshold significantly ↓ PGE2 level in the paw tissue, not in the gastric mucosa. ↓ COX-2 level in the gastric mucosa and paw tissue Activities COX-1 and -2 not effected	
CK	In vivo	CIA-induced DBA/1 mice	CK (224, 56, and 14 mg/kg) per day, 21 days	Significant ↓ in arthritis global assessment and swollen joint count ↑ number of naïve T-cells and ↓ activated T-cells and DCs percentage Inhibited migration and priming of DCs ↓ expressions of CD80, CD86, MHC II, and CCL 21 levels (lymph nodes)	[61]
CK	In vivo	CIA-induced DBA/1 OlaHsd mice	CK (100 μl) once a day (20, 10, and 5 mg/kg/day), 6 weeks (Preventive effect), 4 weeks (Therapeutic effect)	↓ arthritis scores, ↓ serum anti-CII IgG, IFN-γ, and IL-2 ↑ IL-4 levels Non-significant ↓ TNF-α and IL-17 levels ↓ RANKL/OPG and MMP-3/TIMP-1 ratios	[62]
CK	In vivo	Adjuvant-induced arthritis	CK (160, 40, and 10 mg/kg), once daily, 15 days	Significant ↓ in global assessment scores and swollen joint counts ↓ spleen index and hyperplasia of lymph nodes ↓ memory B cells in the spleen ↓ expressions of CD40L (T cells) and CD40 (B cells)	[63]
CK	In vivo	CIA-induced DBA/1 mice	CK (112 mg/kg/day), 24 days	Recovered body weight and ↓ arthritis symptoms, spleen index Inhibited viability and proliferation of lymphocytes ↓ IL-1β, IL-17 and TNF-α and ↑ IL-10 ↓ M1 macrophages and ↑ M2 macrophages; prevented phagocytosis ↑ Gαs expression and inhibited β-arrestin2, NF-κB, TLR4, and Gαi	[64]
CK	In vitro	H_2_O_2_-stimulated MC3T3-E1 cells	CK (0.01-10 μM) with or without H_2_O_2_, 48 h	CK formed hydrogen bonds with IKK ↑ ALP activity, Col-I expressions, and mineralization ↓ ROS and NO production, IL-1β expression	[65]
GNP-CK-CopA3	In vitro	LPS-activated RAW264.7	GNP-CK-CopA3 (10-100 µg/mL), 1 h + LPS (1 µg/mL), 24 h	NO production was inhibited (at 20 and 40 µg/mL) ROS production inhibited-40.4% (20 µg/mL) and 65.05% (40 µg/mL) ↓ levels of TNF-α, iNOS, COX-2, IL-6, and IL-1β Inhibited NF- κB and MAPK signaling pathways	[66]
**Anti-atherosclerosis**					
CK	In vivo	ApoE-/- C57BL/6 Peritoneal macrophages from apoE-/- C57BL/6	CK (9, 3, and 1 mg/kg) one dose per day, 8 weeks. ox-LDL (100 µg/mL) + CK (30, 10, and 3.3 µM)	↓ atherosclerotic plaques (55%) by activating RCT pathway ↓ IL-6, IL-1β, and TNF-α levels ↓ cleaved IL-1β, caspase-1, NLRP3, and NF-kB P65 ↓ inflammasome activity in mice and macrophages ↓ cholesterol ester (10 μM 46.21% and 30 μM 60.24%)	[67]
CK and its derivatives	In vitro	RAW264.7 cells	CK and CK derivatives (30, 10 µM)	Structure 1 ↓ cholesteryl ester contents in foam cells compared to CK ↑ ABCA1 mRNA expression Structure 1 (319%) compared to CK (151%) Structure 1 significantly activated LXRα compared to CK No effect on LXRβ activation	[68]
CK	In vitro	HUVECs	Pretreated with CK (2.5, 1.25, and 0.625 mM), 12 h + ox-LDL (80 mg/mL), 24 h	↓ expressions of IL-6, MCP-1, TNF-α, VCAM1, and ICAM-1 ↓ expression of caspase3, cleaved caspase-3 and cytochrome c and LDH release Reversed mitochondrial membrane depolarization ↑ Bcl2/Bax	[69]
**Anti-diabetic**					
CK	In vivo	HFD fed ICR mice	Injected with STZ (100 mg/kg BW) after 4 weeks + CK (30 mg/kg), 4 weeks	↓ blood glucose levels, improve glucose tolerance ↓ PGC-1α expressions and inhibited PEPCK, G6Pase expressions Improved AMPK phosphorylation	[70]
	In vitro	HepG2 cells	CK (8, 4, and 2 μM), 24 h	Dose-dependent inhibition of hepatic glucose production ↓ PEPCK protein level and ↑ AMPK phosphorylation	
CK and Rb1	In vivo	Epididymal adipose tissue from ICR mice	Glucose treatment (high concentration), 24 h + CK (10 μM) and Rb1 (10 μM)	↓ROS production and ERS ↓ phosphorylation of PERK and IRE1a ↓ activation of NLRP3 inflammasome and ↓ IL-1β, IL-6 production ↓ IRS-1 phosphorylation at a serine residue ↑ IRS-1 phosphorylation at tyrosine residue ↑ PI3K activity and Akt phosphorylation	[71]
CD-CK conjugate	In vivo	Alloxan-induced diabetic zebrafish model	CK and CD-CK (15, 10, 7.5, 5, 2.5, 1, 0.5, 0.1, and 0.05 μM), 2 days	Good recovery of pancreatic islets by CD-CK compared to CK CD-CK showed less toxic (LC_50_ = 20.68 μM) than CK (LC_50_ = 14.24 μM)	[72]
CK conjugate with beta-cyclodextrin	In vivo	HFD-induced C57BL/6 mice	CK (40, 20, and 10 mg/kg/day), 8 weeks	↑ body weight (6th week) ↓ fasting glucose, BUN, creatinine, and urine protein ↓ ROS production and Nox1, Nox4 expressions↓ expressions of NLRP3, Caspase-1, ASC, IL-1β, TNF-α, and IL-18 CK treatment reduced the activation of the p38 MAPK signaling pathway	[73]
	In vitro	High glucose-treated HBZY-1 cells	CK (50, 40, 20, and 10 μM), 48 h	↓ proliferation of HBZY-1 cells ↓ NLRP3, Caspase-1, and ASC levels	

* ex vivo, ST, study type; CK, compound K, SVP, sodium valproate; SD, Sprague-Dawley; LCK, low CK; GCK, ginsenoside CK; MCK, middle CK, HCK, high CK; AST, aspartate transaminase; ALT, alanine aminotransferase; ALP, alkaline phosphatase; TG, triglyceride; ALB, albumin; CAT, catalase; SOD, superoxide dismutase; GPx, glutathione peroxidase; GSH, glutathione; MDA, malondialdehyde; HFD, High fat diet; γ-GT, gamma-glutamyl trans peptidase; CHOL, total cholesterol; FCHOL, free cholesterol; LDL, low density lipoprotein; HDL, high density lipoprotein; TIMP-1, tissue inhibitors of metalloproteinase-1; OLETF, otsuka long-evans tokushima fatty; FAS, fatty acid synthase; SREBP-1c, sterol regulatory element-binding protein-1c; CPT-1, carnitine palmitoyltransferase-1; PPAR-α, peroxisome proliferator-activated receptor-alpha; AMPK, 5′ AMP-activated protein kinase; TC, total cholesterol; TNF-α, tumor necrosis factor alpha; IL, interleukin; α-SMA, alpha smooth muscle actin; ACCα acetyl CoA carboxylase alpha; CYP2E1, cytochrome P450 2E1; p-JNK, phospho c-Jun N-terminal kinase; LPS, lipopolysaccharide; iNOS, inducible nitric oxide synthase; AOX1, aldehyde oxidase 1; Akt, protein kinase B; BIOGF1K, CK and F1; NO, nitric oxide; DPPH, 2,2-diphenyl-1-picrylhydrazyl; IFN-β, interferon-beta; NF-kB, nuclear factor-kB; IRF3, interferon regulatory factor 3; IKK, inhibitor of kB kinase; TBK1, TANK-binding kinase 1; COX-2, cyclooxygenase-2; AP-1 (also known as c-jun), activator protein-1; MAPKs, mitogen-activated protein kinases; MAPKKs, MAPK kinases; BSA, bovine serum albumin; NP, nanoparticles; SPIONs, superparamagnetic iron oxide nanoparticles; ROS, reactive oxygen species; SGLT, sodium-glucose linked transporter or sodium-dependent glucose cotransporters; PGE2, prostaglandin E2; CIA, collagen-induced arthritis; DCs, dendritic cells; CD, cluster of differentiation; MHC, major histocompatibility complex; CCL21, chemokine (C-C motif) ligand 21; CII IgG, type II collagen immunoglobulin G; RANKL, receptor activator of nuclear factor-κB ligand; OPG, osteoprotegerin; MMP, matrix metalloproteinase; TLR4, Toll-Like receptor 4; Gαis, G(i,s) protein subunit alpha; Col-I, type I collagen; GNPs, gold nanoparticles; apoE, apolipoprotein E; ox-LDL, oxidized low density lipoprotein; RCT, reverse cholesterol transport; NLRP3, NOD-like receptor protein-3; ABCA1, ATP-binding cassette transporter A1; LXR, liver X receptor; HUVECs, human umbilical vein endothelial cells; MCP-1, monocyte chemoattractant protein-1; VCAM-1, vascular cell adhesion molecule-1; ICAM-1, intercellular adhesion molecule-1; LDH, lactate dehydrogenase; Bcl2, B-cell lymphoma-2; Bax, B-cell lymphoma 2 (BCL-2)-associated X protein; ICR, imprinting control region; STZ, streptozotocin; PGC-1α, proliferator-activated receptor-γ coactivator-1 alpha; PEPCK, phosphoenolpyruvate carboxykinase; G6Pase, glucose-6-phosphatase; ERS, endoplasmic reticulum stress; IRE1, inositol-requiring enzyme 1; PERK, protein kinase-like ER kinase; IRS-1, insulin receptor substrates -1; PI3K, phosphatidylinositol 3 kinase; BUN, blood urea nitrogen; Nox, NADPH oxidase; ASC, apoptosis-associated speck-like protein containing a CARD.

Similarly, Choi et al. showed the ameliorating effects of GBCK25 (fermented ginseng, rich in CK) on nonalcoholic steatohepatitis (NASH). They found that GBCK25 was capable of downregulating cytochrome P450 2E1 (CYP2E1) levels alongside reduced activation of cellular c-Jun N-terminal kinase (JNK) [55]. These findings indicate that CK can be used for liver disease prevention and/or treatment. 

### 4.2. Anti-Inflammatory 

From previous studies, the anti-inflammatory activity of CK was ascribed to decreased synthesis of pro-inflammatory cytokines ((interleukin (IL)-6, IL-1β, and tumor necrosis factor-alpha (TNF-α)), cyclooxygenase-2 (COX-2), and inducible nitric oxide synthase (iNOS) [11]. However, recent studies have strengthened our understanding of the mechanistic implications at molecular and cellular levels (Table 3). In one study, CK attenuated NF-κB by modulating the Akt1-mediated inflammatory gene expression in LPS-induced macrophages [2]. Compound K-rich fraction (BIOGF1K), consisted of 3.2 g of CK and 1.5 g of saponin F1, and was examined for its anti-inflammatory activity. Compound K-rich fraction has down-regulated LPS-stimulated nitric oxide (NO) production in RAW264.7 cells. Furthermore, expressions of iNOS and IFN-β were reduced by suppressing stimulation of NF-κB and interferon regulatory factor 3, respectively. The inhibitory mechanism of BIOGF1K was due to the blockage of an inhibitor of kB kinase (IKK) and TANK-binding kinase 1 (TBK1), leading to reduced production of NO and IFN-β [56]. Likewise, in another study BIOGF1K inhibited COX-2 and iNOS mRNA expressions in LPS-induced RAW264.7 cells. Mechanistically, BIOGF1 K blocked activation of activator protein-1 (AP-1) pathway by targeting mitogen-activated protein kinases (MAPKs) such as ERK (extracellular signal-regulated kinase) and p38, and MAPK kinases (MAPKKs) such as MAPK/ERK kinase 1/2 and MAPK kinase 3/6 [57]. Together, these findings indicate that BIOGF1 K plays a protective role in macrophage-mediated inflammatory responses. In addition, the use of CK as BSA-CK NPs [39] and CK-conjugated superparamagnetic iron oxide nanoparticles [58], has been shown to have anti-inflammatory activity against RAW 264.7 cells induced by LPS. In another study, CK-mediated modulation of sodium/glucose cotransporter one via the epidermal growth factor receptor (EGFR) pathway was found to reduce intestinal inflammation [59]. 

Inflammation commonly follows pain. In this line, the effect of CK on inflammation and pain was represented using *in vivo* models of xylene-induced ear swelling, and paw oedema stimulated with carrageen. The anti-inflammatory and pain-reducing effects of CK were due to the decreased production of prostaglandin E2 by downregulating COX-2 expression (Table 3) [60]. Referring to arthritis, the attenuating role of CK has been shown by inhibiting the production of inflammatory cytokines, suppressing T-cell activation, inhibiting the multiplication of B-cells, macrophages regulation, and reducing the level of autoantibodies [11]. Among T cells, the potential mechanism of CK treatment involves suppression of dendritic cells (DCs) priming of T-cell activation, suppression of chemokine CCL21 (with receptor CCR7) associated with DC movement and signaling between T cells and DCs in collagen-induced arthritis animal model. Notably, a positive correlation (R^2^ = 0.9830, *p* = 0.0009) was found in percentages of activated T-cells and DCs, while a negative correlation (R^2^ = 0.8348, *p* = 0.03) in percentages of naïve T cells and DCs [61]. In another study, CK suppressed humoral immune response of T helper type 1 (Th1) cells and significantly suppressed expressions of matrix metalloproteinases (MMP)-3 and-13 and receptor activator of NF-κB ligand (RANKL) [62]. Concerning effects on B cells, CK was described as reducing the percentage of memory B cells. Authors suggested that the reduction in memory B cells may be dependent upon T-cells [63]. Previously, CK displayed anti-arithic effects on multifunctional macrophages by reducing the development of pro-inflammatory cytokines. In a recent study, however, the function of CK was shown to inhibit β-arrestin2, thus hindering the transition of macrophages from type M1 to type M2 [64]. The protective role of CK was also reported against osteoarthritis using in vitro and silico studies. Compound K displayed high binding affinity to a cytokine-activated kinase (IKK) compared to other ginsenosides as revealed in the molecular docking analysis. Thus, the anti-osteoarthritic effect of CK was due to inhibition of IKK activity in vitro [65]. Interestingly, in a recent study, GNPs were made intracellularly using *Gluconacetobacter liquefaciens* kh-1 (a probiotic strain) and used for synthesizing peptide (CopA3) conjugated nanoparticle (GNP-CK-CopA3) hybrids. Compound K, as peptide-nanoparticle hybrids showed anti-inflammatory effects by inhibiting the activation of NF-kB and MAPK signaling pathways [66]. 

### 4.3. Anti-Atherosclerosis 

Atherosclerosis is well known to be an inflammatory disease; the anti-inflammatory effects of CK are more or less directly linked to its anti-atherogenic effects. In terms of anti-atherosclerosis, an important feature of CK was found to be associated with liver X receptor alpha (LXRα) (Table 3). Targeting LXRα, a study showed that CK treatment resulted in a dose-dependent reduction of atherosclerotic plaques by activating the reverse cholesterol transport (RCT) pathway, reducing inflammatory cytokines, and inhibition of inflammasome activity with LXR activation in apoE-/- C57BL/6 mice. Compound K triggered the RCT pathway by upregulating ATP-binding cassette transporter (ABC) A1, ABCG1, LXRα, ABCG5, and ABCG8. In addition, CK supplementation increased the cholesterol efflux and reduced the inflammasome activity in peritoneal macrophages of mice [67]. Another research demonstrated the use of CK and its derivatives in the activation of LXRα. The study documented the synthesis of six CK derivatives by adding short-chain fatty acids into the carbohydrate chain of CK at different locations. Effects on the foam cell model were evaluated, and the biological activities of all derivatives were found to be at par or better than their parent CK. All derivatives were capable of activating LXRα. Compound K derivative 1 displayed the best potency amongst all [68]. 

Similarly, it was found that the CK prevents inflammation and apoptosis in human umbilical vein endothelial cells, induced through oxidized low-density lipoprotein (ox-LDL). In endothelial cells, lectin-like oxidized low-density lipoprotein receptor-1 (LOX-1) uptakes ox-LDL leading to pro-inflammatory effects. Compound K decreased LOX-1 expression and inhibited the nuclear translocation of NF-kB, and phosphorylation of JNK and p38 [69]. The results indicate that CK [67,69] and its derivatives [68] have the anti-atherosclerosis effect. 

### 4.4. Anti-Diabetic 

Ginsenosides play an important anti-diabetic role by modulating insulin resistance, regulating lipid and glucose metabolism, protecting from the inflammatory response, and oxidative stress. In this line, a study showed that CK administration suppressed liver gluconeogenesis by inhibiting glucose-6-phosphatase and phosphoenolpyruvate carboxykinase expressions in HFD-fed ICR mouse model and HepG2 cell line. Meanwhile, the expressions of hepatocyte nuclear factor 4 alpha, peroxisome proliferator-activated receptor 1-alpha, and forkhead transcription factor O1 were decreased while AMPK phosphorylation was increased significantly [70]. Furthermore, the management of insulin resistance is essential for controlling diabetes. In this line, CK was found to be able to inhibit inflammation and modulate insulin resistance in adipose tissue by repressing the activation of NOD-like receptor family, pyrin-containing protein 3 (NLRP3) associated with endoplasmic reticulum stress (ERS) (Table 3) [71]. In another study, beta-cyclodextrin-conjugated CK (β-CD) was used to modulate diabetes against an alloxan-induced zebrafish model. The recovery of affected pancreatic islets in CD-CK conjugate was significantly higher (EC_50_ = 2.16 μM) than in CK (EC_50_ = 7.22 μM) [72]. Furthermore, the protective effect of CK on diabetic nephropathy in HFD/ streptozotocin-induced mice has been demonstrated through significant reduction of oxidative stress and down-regulating expressions of NADPH oxidase (Nox)-1 and-4 proteins. Additionally, the reactive oxygen species (ROS)-mediated activation of the inflammasome assembly was reduced, and renal p38 MAPK phosphorylation was inhibited (Table 3) [73].

### 4.5. Anti-Cancer 

The promising anti-cancer activity CK has been previously identified in various types of cell lines, including lung carcinoma, leukemia, breast cancer, colorectal cancer, prostate cancer, gastric carcinoma, nasopharyngeal carcinoma and pulmonary adenocarcinoma [11,74]. Among recent findings (Table 4), a study documented the suppressing effect of CK on COX-2 and Arg-1 genes linked to immunosuppression, apoptosis, and pro-inflammatory cytokines production by myeloid-derived suppressor cells (MDSCs) from the xenografted colorectal (CT26) cancer mice. Compound K could act as a promising therapeutic molecule by targeting MDSCs [75]. Another study elucidated the inhibitory action of CK on the development and metastasis of glioblastoma cell lines (U87MG and U373MG). The effects were due to cell cycle arrest, decreased cyclins (D1 and D3) levels, apoptosis through nuclear condensation, activation of apoptotic enzymes, increased production of ROS, and the depolarized potential of the mitochondrial membrane. The anti-proliferative effect was due to the blockage of the phosphatidylinositol three kinase (PI3K)/Akt signaling pathway in glioblastoma [76]. Likewise, CK was found to block glycogen synthase kinase 3β signaling [77] and the PI3K/Akt signaling pathway [78] in breast cancer cells (MCF-7). Additionally, the combined CK and cisplatin had better effects than either molecule alone [78]. A later study gave in vivo evidence of CK’s protecting effects against hormone-independent breast cancer by degrading cyclin D1 protein [79] (Table 4). Recently, Li et al. synthesized ester derivatives (1c, 2c, 3c) of M1, and found that among all, compounds 2c, 3c had an effective growth inhibitory effect on MCF-7 cells [80]. Another study shed light on the biological mechanism of CK against breast cancer using SKBR3 cells. Compound K displayed anti-cancer effects in SKBR3 cells by enhancing apoptosis through downregulation of Akt-1. In addition, CK was found to reduce invasion and metastasis [81]. 

Compound K inhibited proliferation, augmented autophagy, and apoptosis of non-small cell lung cancer (NSCLC) (A549 and H1975) cells through the mammalian target of rapamycin (mTOR)/AMPK and JNK signaling pathways (Table 4) [82]. Furthermore, the suppression of the growth of NSCLC cells was studied by targeting the metabolism of glucose. Compound K suppressed the levels of hypoxia-inducible factor 1-alpha and its downstream glucose transporter1 gene [83]. Another study showed the anti-cancer effect of CK against HepG2 cells and xenografted (HepG2) BALB/c nude mice. Compound K resulted in cell cycle arrest, blocked cell cycle progression, and apoptosis induction by modulating B-cell lymphoma 2 (Bcl2) to Bcl2 associated X (an apoptosis regulator) ratio in HepG2 cells. Furthermore, a substantial reduction in tumor proliferation was observed in the CK-supplemented mice group [84]. Also, CK induced apoptosis and ERS in liver cancer cells and xenografted mice by modulating signal transducers and activators of transcription-3 (STAT3) activation [85]. Another study showed for the first time that CK targeted annexin A2, which leads to inhibition of NF-κB [86]. Compound K enhanced ERS and calcium release by ryanodine receptors leading to apoptosis in lung cancer cells of humans. In particular, the use of an ER stress inhibitor (4-phenylbutyrate) enhanced CK- mediated apoptosis [87]. Another research provided the first proof that the CK usage results in the TNF-related apoptosis-inducing ligand (TRAIL) sensitization in TRAIL-resistant HT-29 cells and potentiated TRAIL-stimulated apoptosis in HCT116 by autophagy-linked death receptor (DR) 5 stimulation. The upregulated expression of DR5 was dependent upon ROS mediated JNK-autophagy-activation and CCAAT/enhancer-binding protein (C/EBP) homologous protein/p53 pathway (autophagy-independent) [88]. In neuroblastoma cells, CK enhanced ROS-linked apoptosis and impaired the autophagic flux. In addition, CK with chloroquine (combination approach) stimulated apoptosis in cell line and mouse models and may, therefore, be a potential approach for treating neuroblastoma [16]. The protective role of CK was also investigated against glioma (inveterate brain tumor). Compound K was observed to inhibit the stromal cell-derived growth factor 1 migration of C6 glioma cells by controlling protein kinase C alpha, ERK1/2, and MMP signaling molecules (Table 4) [89]. Recent studies showed the bioactivity of CK against human osteosarcoma cell (MG63 and U2-OS) lines.

**Table 4:** Anti-cancer, neuroprotection, anti-aging/skin protection, and other activities of CK and its derivatives.

Material	ST	Model	Treatments	Major Findings	Ref.
**Anti-cancer**					
CK	In vivo	Balb/c mice with CT26 tumor cells		↓expression of Cox-2 and Arg-1 ↓ productions of IL-1β, IL-6, and IL-17 ↓ CT26 tumor growth	[75]
CK	In vitro	U87MG and U373MG cells	CK (50, 20, and 10 µM), 72 h	Significant growth reduction of target cells and inhibited cells mobility and invasion G0/G1 phase arrest for U87MG (80.7%) and U373MG (77.3%) ↑ apoptosis Negative regulation of PI3K/Akt/mTOR signaling pathway	[76]
CK	In vitro	MCF-7 cells	CK (70, 50, 30, and 10 µM), 24 h	Inhibited proliferation dose-and time-dependently ↓ expressions of GSK3β, cyclin D1, and β-catenin	[77]
CK	In vitro	MCF-7 cells	CK (50 mmol/L) or cisplatin (10 mg/L), alone or in combination, 24–96 h	Anti-proliferation activity: CK (19.18 ± 2.25), cisplatin (21.34 ± 2.84), and both (43.37 ± 5.62) ↑ apoptosis in the combined treatment compared to individual treatments	[78]
CK	In vivo	Xenograft nude mice	CK (1 or 0.2 mg/kg), every other day, 3 weeks	Reduction in the tumor weight	[79]
	In vitro	MCF10DCIS.com and MCF10CA1a	CK (20, 10 μM), 24, 48, and 72 h	↓ viability in dose-and time-dependently ↑ cell cycle blockage ↓ cyclin D1 production and ↑ cyclin D1 degradation	
M1 and its derivatives	In vitro	MCF-7 and MDA–MB–231 cells	M1, 1c, 2c, and 3c (100, 50, 25, and 1 μM)	Derivatives 2c and 3c showed good inhibitory effects 80% inhibition for MCF-7 (lower con.) For MDA-MB-231, better effects on higher concentration Derivatives 2c and 3c changed membrane permeability and promoted apoptosis of MCF-7	[80]
CK	In vitro	SKBR3 cells	CK (0–50 μM), 3–24 h	↑ anti-proliferative and apoptotic activities ↑ levels of cleaved caspase-7, -8, and caspase-9 ↓ Bcl2 levels and AKT-1 levels, no effect on AKT-2 levels	[81]
CK	In vitro	A549 and H1975	CK (20 μg/mL), 24 h	↑ anti-proliferative and apoptotic activities ↑ beclin-1 protein level ↓ p-JNK/JNK, p-c-Jun/c-Jun, LC3II/LC3I and p62 levels ↑ levels of caspase-3 and cleaved PARP in both cells AMPK/mTOR, JNK signaling pathways activated	[82]
CK	In vitro	NSCLC		Dose-dependent anti-proliferative effect Inhibited expression of PDK1, HK II, and LDHA Inhibited expressions of HIF-1α and GLUT1	[83]
CK	In vivo	Xenografted BALB/c mice	CK (10 mg/kg/day)	Reduced tumor volume and ↓ tumor weight (49.4%)	[84]
	In vitro	HepG2 cells	CK (20, 10, 5, and 2.5 µmol/L), 48 h	↓viabilities of HepG2 cells in dose-and time-dependently and ↑ apoptosis ↑ cell arrest at 5 µmol (68.61 ± 2.91%) and l0 µmol (l78.29 ± 2.57%) ↓ expressions of cyclin D1 and CDK-4 ↑ expressions of cleaved-caspase-3, -9, Bax, p21^Cip1^ and p27^Kip1^ ↓ Bcl-2 and PARP (inactive)	
CK	In vivo	SMMC-7721 cells injected BALB/c nude mice	CK (20, 10, and 5 mg/kg/day), 15 days	Dose-dependent inhibition of tumor Significant ↓ in body weight of mice (20 mg/kg) ↓ p-STAT3 levels	[85]
	In vitro	HepG2 and SMMC-7721	CK (60, 40, and 20 µM) 48 h	↑apoptosis and ERS in cell lines ↓ DNA-binding ability of STAT3 ↓ p-STAT3 levels ↑ERS markers (CHOP and GRP78) expressions PERK and IRE1 signaling pathways activated	
CK	In vitro	HepG2 cells	CK (6 µM), 12 h	↓ interaction and of colocalization (nucleus) of p50 and annexin A2 NF- κB signaling pathways activation inhibited and ↓ downstream genes expressions	[86]
CK	In vitro	A549 and SK-MES-1	CK (15, 10, and 5 μM), 48 h and 15 μM, 6, 12, 24, 36, or 48 h	IC_50_ for viabilities of A549 (17.78 μM) and SK-MES-1 (16.53 μM) ↑ caspase 12 dependent apoptosis Induced ERS by ↑ p-eIF2α expressions and protein levels of XBP-1S, GRP78(BiP), and IRE1α ↑ intracellular calcium levels and m-calpain activities	[87]
CK	In vitro	HT-29 and HCT116 cells	CK (50 or 20 μM), 24 h	↓expressions of Mcl-1, survivin, Bcl-2, XIAP, and cFLIP ↑ expressions of tBid, Bax, and cytochrome c, and DR5 ↑ in LC3-II and Atg7 levels and expressions of p53 and CHOP ↑ JNK phosphorylation	[88]
CK	In vivo	SK-N-BE(2) injected BALB/c nude mice	CK (30 mg/kg) and chloroquine (50 mg/kg), 3 times/week/60 days	↑TUNEL-positive cells and caspase-3 expression Compared to chloroquine, CK and CK+ chloroquine significantly reduced tumor size ↑ inhibition in the combination approach	[16]
	In vitro	SK-N-BE(2) and SH-SY5Y cells	CK (20, 15, 10, 5 and 2 µM), 24 h	↑ cell cycle arrest (at sub G1 phase), ROS production and P21 protein level a ↑ caspase-dependent apoptosis Induced early phase autophagy by ↑ BECN, Atg7, and LC3B expressions Inhibited late phase autophagy	
CK	In vitro	SDF-1 induced C6 glioma cells	CK (10, 3, 1, 0.3, 0.1, and 0.03 μM), 24 h	CK abridged scratch wound-healing and inhibited C6 cells migration ↓ phosphorylation of downstream targets PKCα (SDF-1 pathway) and ERK1/2 (CXCR4 pathway)	[89]
CK	In vitro	MG63 and U2-OS cells	CK (30, 25, 20, 15, 10, and 5 µM), 3 days	Anti-proliferative effect against osteosarcoma cells (IC_50_ = 20 µM for 3 days) ↑apoptosis rate CK (20 µM): U2-OS (17.66 ± 1.37%), MG-63 (24.16 ± 2.25%) Suppressed invasion and migration Blocked PI3K/mTOR/p70S6K1 signaling pathway ↑PTEN levels in both cells ↓p-AKT and p-mTOR in both cells ↓expressions of p-mTOR, p-mTOR/mTOR ratio and p70S6K1 in U2-OS cells treated with RAD001 (mTOR inhibitor)	[90]
GCKT-liposomes	In vivo	Athymic nude mice	GCK (15 mg/kg), GCKT-liposomes (15 mg/kg)/ 5 times every 3 days	GCKT-liposomes group, ↓mean tumor size from 219.0 ± 17.0 mm^3^ to 45.8 ± 3.2 mm^3^ slow ↑ in body weight in the initial days later no change	[33]
	In vitro	A549	GCK + GCKT-liposomes different concentrations, 24 h	IC_50_, GCKT-liposomes (16.3 ± 0.8 μg/ml) and CK (24.9 ± 1.0 μg/ml) No cytotoxicity to A549 with T-liposomes alone	
CK-M (TPGS/PEG-PCL+CK)	In vivo	Male athymic nude mice	CK and CK-M (15 mg/kg) once every 3 days, 15 days	Tumor volume after treatment CK-M (2.67 ± 0.88), CK (4.27 ± 0.35) CK-M ↓ tumor growth (79.12 ± 0.60 to 52.04 ± 4.62%) Bodyweight: CK-M group (25.02 ± 2.42), control (22.83 ± 1.83) low toxicity of CK-M to the mouse model	[36]
	In vitro	A549 and PC-9 cells	CK or CK-M (100, 50, 25, 12.5, 6.25, and 3.125 μg/mL), 24 h	IC_50_ for A549: CK (21.97 ± 1.50 μg/mL) CK-M (25.43 ± 2.18 μg/mL) IC_50_ for PC-9: CK (14.46 ± 1.24 μg/mL) CK-M (18.35 ± 1.90μg/mL) ↑CK-M uptake by A549, PC-9 cells ↑ apoptosis ↓ inhibited tumor cell invasion and metastasis Regulated Bcl-2, Bax, MMP-2, and Caspase-3 levels	
CK-AP/TPGS	In vivo	Nude mice	CK-AP/TPGS (30 mg/kg) every 3 days until the 12^th^ day	Maximum anti-tumor effect (66.24 ± 8.77%) by CK mixed micelles at 15th day low toxicity to kidney and liver ↑ apoptosis of tumor tissue ↑ Bax/Bcl-2 ratio (7.25-times) ↑ cellular uptake and tumor targeting	[37]
	In vitro	A549 cells	CK-AP/TPGS and CK (80, 40, 20, 10, and 5, μg/mL, 24 h	CK mixed micelles had a better effect on cell cycle arrest at G0/G1 phase than free CK IC_50_ for A549: free CK (16.11 ± 1.23 µg/mL) and CK mixed micelles (10.29 ± 1.1 µg/mL) ↑ apoptosis, A549: CK mixed micelles (45 ± 5.25%) and CK (17.28 ± 2.25%)	
CK PC/DP micellar system	In vivo	Xenografted nude mice	CK or CK mixed micelles (30 mg/kg) every 3 days for 12 consecutive days	No damage to liver and kidney Significant apoptosis of tumor tissue ↑ Bax/Bcl-2 ratio ↑ expressions of caspase-3, -8, -9 and PARP	[38]
	In vitro	A549 cells	CK or CK mixed micelles (12.15 μg/mL), 24 h	IC_50_ for A549: CK (18.31 μg/mL) and CK mixed micelles (12.15 μg/mL) Effective cell cycle arrest at G1 by CK PC/DP compared to CK Highest apoptosis rate in CK PC/DP compared to CK	
BSA-CK NPs	In vitro	HaCaT, HepG2, A549, HT29 cancer cells. LPS- induced RAW264.7 cells	CK and BSA-CK (20, 15, 10, 5, and 1 μM), 24 h	Improved anti-cancer ability of BSA-CK NPs compared to CK Higher ↓ in NO production by BSA-CK NPs	[39]
DCY51T AuCKNps	In vitro	A549, HT29, AGS and RAW264.7 cells	DCY51T AuCKNps 0.1, 1, 5, 10, 15, and 20 μM Phototherapy- NPs+ AGS + 1 or 5 mg/mL, 24 h + laser at 800 nm, 10 min.	↑cytotoxicity for A549 and HT29 compared to CK ↑apoptosis after laser treatment in AGS	[40]
CK + chitosan NPs	In vitro	HepG2 cells	CK and CK-NPs (3.125, 6.25, 12.5, 25, and 30 μg/mL), 24 h	At 30 μg/mL, the apoptotic cell percentage, CK (39.02 ± 0.42%) and CK-NPs (47.57 ± 1.65%)	[41]
GK-OCMC NPs	In vitro	PC3 cells	CK and GK-OCMC NPs (30 μg/mL)	↑ apoptosis by GK-OCMC treatment ↑ levels of caspase-3 (29.93%) and caspase-9 (20.78%) compared to GK treatment.	[42]
APD-CK micelles	In vitro	HepG2 and Huh-7 cells	CK (30, 20, 10, 5, and 2.5 μg/mL), 24 h and 48 h	Time-dependent and dose-dependent cytotoxic effects of APD-CK ↑expressions of PARP, caspase-3, and -9 in HepG2 cells by APD-CK micelles	[43]
Parthenolide/ CK tLyp-1 liposomes	In vivo	Nude mice	5 mg/kg, 24 h	Strong tumor inhibition with parthenolide/ CK tLyp-1 liposomes than combined	[91]
	In vitro	A549	Parthenolide (1.5 µg/mL) + CK (30 µg/mL) in 5:1 ratio	↑ mitochondrial apoptosis: CK (8.2%), parthenolide (11.8%), CK+ parthenolide (34.7%), Parthenolide/ CK tLyp-1 liposomes (56.7%) ↑ROS levels: CK (3.7%), parthenolide (5.8%), CK+ parthenolide (24.6%), Parthenolide/ CK tLyp-1 liposomes (28.7 %) Marked structural changes in mitochondria and impaired mitochondrial membrane potential	
CKGal	In vitro	AGS, B16F10, HeLa, and U87MG	CKGal, CK, F12, and Rh2 each at (200, 100, 50, 25, 12.5, 6.25 µmol), 72 h	↓ cell viability: U87MG (13.7%), AGS (8.7%), B16F10 (2.6%), and HeLa (7.3%) IC_50_ CKGal: HeLa (40.38 µmol), U87MG (40.38 µmol), B16F10 (22.4 µmol), and AGS (4.487 µmol) cells	[92]
**Neuroprotection**					
CK from RG	In vitro	Glutamate-induced HT22 (hippocampal) cells	CK (8, 4, 2, and 1 μM), 12 h	↓glutamate-induced cytotoxicity Induced Nrf2 growth in the nucleus ↑ expressions of HO-1, NQO1, and GR and ↓ Nrf2 and Keap1 expressions	[93]
	In vivo	Scopolamine-induced C57BL/6J mice	CK (10, 5, and 1 mg/kg) daily, 2 weeks	Restored memory and cognitive functions Modulated Nrf2-mediated cognitive functions	
CK	In vivo	Diabetic db/db mice	CK (10 mg/kg) per day, 12 weeks	Improved cognitive dysfunction, behavioral impairment, glucose tolerance and insulin sensitivity, and dyslipidemia ↓ fasting glucose levels and ↓ IL-1β, TNF-α, and IL-6 in the hippocampus ↓MDA levels ↑ SOD and GSH-Px activities ↓TXNIP, NLRP3 inflammasome, ASC, cleaved IL-1β, and cleaved caspase-1 ↓ CHOP, BiP p-PERK, p-IRE1α, and total ATF6 (Ameliorated ERS)	[94]
CK	In vivo	SD rats Cortical neurons from C57BL/6 mice	Morphine (26 nmol/10 mL) per h, CK (10 mg/10 mL/h), 7 days + naloxone (10 mg/kg), 6 h CK (5, 1, and 0.1 mM), 30 min + morphine (1 mM), 6 h	↓escape behavior and teeth chattering ↓p-ERK, p-NR1 ↓NR1, p-NR1 levels No significant effect on ERK	[95]
CK	In vivo	SD rats	CK (120, 160, and 80 mg/kg), twice a day at 12 h interval, 5 days followed by lithium chloride-pilocarpine or PTZ	PTZ-induced behavioral seizures ↓reduced the seizure intensity and duration and prolonged latency (High dose) Lithium chloride-pilocarpine-induced behavioral seizures ↓reduced the seizure intensity and prolonged latency (High dose) ↑ GABA levels and GABAARa1 and KCC2 expressions ↓NKCC1 expressions	[96]
CK	In vivo	Kunming mice	CK (30, 10, and 3 mg/kg) since 8 to 14 day after partial hepatectomy	Improved MWM test scores of POCD mice ↓ TNF-α, IL-1β, and LDL-C serum levels ↑ HDL-C levels In Hippocampal tissues: ↓ IL-1β, TNF-α, and NF-κB P65 ↑ downstream targets of LXRα- ABCG1, ABCA1, and apoE	[97]
CK	In vivo	Memory-impaired ICR mice induced with scopolamine hydrobromide	CK1 (CK 20 mg/kg + SCOP 2 mg/kg); CK2 (CK 40 mg/kg + SCOP 2 mg/kg), daily, 2 weeks	↑memory function ↓ neuronal apoptosis and its morphology restored Inhibited expression of Amyloid β ↑ SOD and GPx levels and reduced ↓ MDA levels Activated Nrf2/Keap1 signaling pathway	[98]
CK	In vitro	Amyloid β peptide treated HT22 cells	CK (10, 5, and 2.5 μM), 24 h	↑ survival rate and restored growth and morphology of HT22 cells ↓apoptosis and expression of amyloid β peptide ↑ expressions of GLUT3, GLUT1, IRS2, and IDE ↓ expressions of CDK5, GSK3β, and tau	[99]
CK	In vivo	SD rats	CK (200,100, and 50 mg/kg), 8 weeks	↓ cognitive discrepancies in vascular dementia rats at 200 mg/kg Ameliorated neuronal damage Significant ↓ of amyloid β1-42 ↑Akt or protein kinase B activity, involved in the PI3K/Akt pathway leading to ↑GSK3β and IDE levels	[100]
CK	In vivo	Wistar rats	CK (60 and 30 mg/kg/day), 15 days	Significant ↓ in neurobehavioral scores ↓water content in brain tissue at 60 mg/kg/day ↓ brain infarct volume ratio ↑SOD and GSH-Px activities and ↓MDA levels ↓expressions of inflammatory molecules	[101]
CK	In vivo	Kunming mice SD rats	CK (30, 10, and 3 mg/kg), daily once, 4 weeks CK (30, 10, and 3 mg/kg), daily once, 2 weeks	Improved depressive-like activities in mice In rats, ↑sucrose preference and body weight Improved food consumption and crossings in CUMS rats ↑dopamine and 5-HT (serotonin) levels and no effect on norepinephrine ↓ expression of neurotransmitter degrading enzymes ↑BDNF, NGF levels and SOD, GPx, and GSH activities	[102]
CK	In vivo	Kunming mice	CK (30, 10 and 3 mg/kg), 4 weeks	Prevented depressive-and anxiety-like behaviors ↓ MDA level and ↑ SOD expression ↓ IL-1β and IL-18 Inhibited expressions of NLRP3 and cleaved caspase-1	[103]
CK	In vitro	Thrombin-induced EnNSCs	CK (10 μM)	Improved sphere-forming ability ↓apoptosis of EnNSCs ↑ proliferation of Ki67-positive EnNSCs cells ↑ neurogenesis of Doublecortin-positive EnNSCs cells Activated LXRα signaling by ↑ expressions of HMGB3 and RBBP7	[104]
	In vivo	Thrombin-induced C57BLC/6	CK (10 mg/kg)	Improved the neurobehavioral function ↑ neurogenesis in cerebral subventricular zone	
CK	In vivo	C57BL/6 mice 2 months and 24 months old treated	CK (15, 10, and 5 mg/kg), 3 days. Last CK dose, EdU treatment for 24 h for cell proliferation. Neuronal survival: Last CK dose, followed by EdU for 3 days sacrificed after 4 weeks	↑EdU-incorporated cells in 2 months’ dose (dose-dependent) and 24 months at 15 mg/Kg ↑number of cells: PCNA labeled/EdU+PCNA labelled and Ki-67/EdU+Ki-67 positive cells ↑new cells survival and their differentiation into neurons (observed in cells labeled with EdU+ NeuN) ↑ BDNF and NT3 levels Induced phosphorylation of Akt and ERK1/2 at 10 mg/Kg (2 months) and 15 mg/Kg (24 months)	[105]
**Anti-aging/skin protection**					
CK	In vitro	HaCaT	CK (0.01-1 μM), 3 h	↑ hyaluronic acid production ↑phosphorylation of ERK and Akt	[106]
CK	In vitro	Pretreated NIH3T3 cells HaCaT cells B16F10 cells	CK (0-10 μM) +UV (30 mJ/cm^2^) irradiation followed by CK, 24 h	↓MMP1 and COX-2 levels Restored collagen (I) level ↑ TGM, FLG, and HAS-1 and -2 (slight) levels ↑ melanin content but no effect on melanin secretion and tyrosinase activity Modulated phosphorylation of IκBα MAPKs, JNK, and ERK	[107]
BIOGF1K	In vitro	UVB-treated (30 mJ/cm2) NIH3T3 cells	BIOGF1K 30, or 15 mg/mL, 24 h	No cytotoxicity, Inhibited apoptosis Repressed morphological changes ↓ melanin secretion and restored sirtuin 1 and type I procollagen levels ↓ levels of MMP-1, MMP-2, COX-2 and ↓ activity of AP-1 and MAPK	[108]
CK	In vitro	HaCaT cells	CK (5 μM), maclurin (15 μM), and maclurin /CK, 24 h	No cytotoxicity to HaCaT cells ↓MMP-1 level in combination than in individual treatments	[109]
CK	In vivo	UV-treated (100 mJ/cm^2^) SKH-1 (hairless) mice DNCB-induced atopic dermatitis	CK (0.3%), daily two times, 2 weeks CK (0.3%), daily two times, 2 weeks	TEWL value: in UVB treated group (85 g/m^2^/h) and CK + UVB group (57 g/m^2^/h) and in DNCB-treated group (65 g/m2/h) decreased to (42 g/m2/h) in CK + DNCB group Improved skin hydration: 37% from 31% (UV-treated) and 28% from 20% (DNCB-treated) CK improved epidermal hyperkeratosis Suppressed skin thickness to 73.5% in the UV model and 50.5% in the DNCB model ↑ SPINK5 levels and ↓ KLK-5 and PAR2 in both models	[110]
	In vitro	HaCaT cells UV-treated (15 mJ/cm^2^)	CK (30, 10, 3, and 1 μM), 24 h	↓ SPINK expression by decreasing KLK-7, -5 and PAR2	
CK	In vivo	Imiquimod (IMQ)-induced psoriasis-like dermatitis C57BL/6 female mice	CK (0.1% and 1%), next three days	CK (1%) suppressed imiquimod-induced keratinocyte proliferation ↓ epidermal thicknesses ↓ RegIIIγ expression in IMQ-treated mouse keratinocytes	[111]
	In vitro	HaCaT cells	CK (2, 1.6, 1.2, 0.8, and 0.4 μg/mL) + IL-36γ (μg/mL)	Dose-dependent inhibition of proliferation No effect on apoptosis CK (0.4 μg/ml) inhibited REG3A expression induced by IL-36γ	
**Others**					
CK	In vivo	UUO C57BL/6 mice after) UUO induction I/R injury with unilateral NX model	CK (30 mg/kg body wt.) therapeutic group (1 day before), preventive group (3 days) + one day after ligation of renal vessels	UUO model, ↓NLRP3 inflammasome activation in kidney↑ribosome-governed activation Prevented renal tubulointerstitial lesions in the kidney ↓TNF-α, IL-6, IL-1β, and MCP-1 in urine Inhibited activation of T-cells and NF-κB Improvement kidney pathology and kidney function in NX + I/R model (Therapeutic effects)	[112]
	In vitro	M-1 under MICP J774A.1 macrophages	CK (10 μM), 30 min CK (0-10 μM), 30 min + LPS, 5.5 h	↓ caspase-1, IL-1β NF-κB p65, and NLRP3 in M-1 cells Suppressed NLRP3 expression by ↓NF-κB activation in macrophages ↓ phosphorylation and activity of STAT3 in activated macrophages	
CK (M1)	In vivo	LPS-induced NZB/WF1 mice	M1 (50 mg/kg)	↓levels of BUN, Cr, albuminuria, and anti-dsDNA autoantibodies ↓glomerulonephritis activity scores ↓IL-1β, TNF-α, IFN-γ, IL-6, MCP-1, and IL-12p70 ↓T-cell proliferation, number of Th cells (expressed IL-4 or IFN-γ), CD3^+^CD69^+^ cells, and CD4^+^CD69^+^ cells	[113]
	In vitro	LPS-treated BMDCs, podocytes	M1 (10 μM), 30 min + with or without LPS (100 ng/mL), 6 h	↓ ROS production and inhibited activation of NLRP3 inflammasome	
CK analogues	In vivo	OVA-sensitized asthmatic mouse	CK and its analogues (20 mg/kg) for 7 days	Comparable anti-asthmatic effects of CK analogues T1, T2, T3, T8 and T12 IgE (ng/mL) value = CK (1501.85 ± 184.66), T1 (1237.11 ± 106.28), T2 (975.82 ± 160.32), T3 (1136.96 ± 121.85), T8 (1191.08 ± 107.59) and T12 (1258.27 ± 148.70)	[114]
CK	In vitro	H9c2 cells	CK (2, 4, and 8 μM), 48 h	↑cell survival and ↓cell damage ↓ROS production and mitochondrial damage ↓ production of phagocytic precursors ↓ Bax/Bcl-2 ratio, cleaved caspase-3 and PARP ↓ p-Beclin-1/beclin-1ratio, Atg5, Atg7, LC3II/I ↑ P62 expression	[115]

CK, compound K; ST, study type; COX-2, cyclooxygenase-2; IL, interleukin; PI3K, phosphatidylinositol 3 kinase; Akt, protein kinase B; mTOR, mammalian target of rapamycin; GSK3β, glycogen synthase kinase 3β; Bcl2, B-cell lymphoma-2; JNK, c-Jun N-terminal kinase; AP-1 (also known as c-jun); LC3, microtubule-associated protein 1A/1B-light chain 3; PARP, poly (ADP-ribose) polymerase; AMPK, 5′ AMP-activated protein kinase; NSCLC, non-small cell lung cancer; PDK1, 3-Phosphoinositide-dependent protein kinase 1; HKII, mitochondrial hexokinase II; LDHA, lactate dehydrogenase A; HIF-1α, hypoxia-inducible factor 1; GLUT, glucose transporter; CDK-4, cyclin-dependent kinase-4; Bax, B-cell lymphoma 2 (BCL-2)-associated X protein; ERS, endoplasmic reticulum stress; STAT3, signal transducer and activator of transcription 3; p-STAT3, phosphorylated-STAT3; CHOP, C/EBP homologous protein; GRP78, glucose-regulated protein-78; PERK, protein kinase-like endoplasmic reticulum kinase; IRE1, inositol-requiring enzyme 1; NF-kB, nuclear factor-kB; p-eIF2α, phospho-eukaryotic translation initiation factor 2 subunit α; XBP-1S, X-box binding protein-1S; Bip, binding immunoglobulin protein; Mcl-1, myeloid cell leukemia 1; XIAP, X-linked inhibitor of apoptosis protein; cFLIP, fas-associated death domain-like IL-1-converting enzyme-inhibitory protein; tBid, truncated BID; DR5, death receptor 5; Atg7, autophagy-related 7; TUNEL, TdT-mediated dUTP nick end labelling; ROS, reactive oxygen species; BECN, Beclin-1; SDF-1, stromal cell-derived growth factor 1; PKCα, protein kinase Cα; ERK, extracellular signal-regulated kinase; CXCR-4, C-X-C chemokine receptor type 4; IC_50_, half maximal inhibitory concentration; p70S6K1; ribosomal protein S6 kinase β 1; PTEN, phosphatase and tensin homolog; GCK, ginsenoside CK; GCKT, GCK with TPGS; TPGS, D-α-tocopheryl polyethylene glycol 1000 succinate; PEG, polyethylene glycol; PCL, polycaprolactone; MMP, matrix metalloproteinase; AP, ascorbyl palmitate; PC, phosphatidylcholine; DP, 1,2-distearoyl-sn-glycero-3-phosphoethanolamine polyethylene glycol 2000; BSA, bovine serum albumin; NP, nanoparticles; NO, nitric oxide; OCMC, O-carboxymethyl chitosan; APD-CK, A54-PEG-DA−OCMC polymer CK-loaded micelle; ROS, reactive oxygen species; tLyP-1, truncated form of the cyclic tumor-homing peptide LyP-1; RG, red ginseng; Nrf2, nuclear factor (erythroid-derived 2)-like 2; HO-1, heme oxygenase-1; NQO1, NAD(P)H dehydrogenase [quinone] 1; GR, glutathione reductase; Keap1, Kelch-like ECH associated protein 1; TNF-α, tumor necrosis factor alpha; MDA, malondialdehyde; iNOS, inducible nitric oxide synthase; MAPKs, mitogen-activated protein kinases; SOD, superoxide dismutase; GSH, glutathione; TXNIP, thioredoxin-interacting protein; NLRP3, NOD-like receptor protein-3; ASC, apoptosis-associated speck-like protein containing a CARD; p-IRE1α, phospho-IRE1 alpha; ATF6, activating transcription factor 6; SD, Sprague-Dawley; p-PERK, phospho-PERK; p-NR1, phospho-N-methyl-D-aspartate acid receptor subunit 1; p-ERK, phospho-ERK; PTZ, pentylenetetrazole; GABA, gamma amino-butyric acid; GABA_A_Rα1, GABA type A receptor subunit alpha1; KCC2, K-Cl cotransporter isoform 2; NKCC1, Na-K-2Cl cotransporter isoform 1; MWM, Morris water-maze; POCD, post-operative cognitive dysfunction; LDL-C, low density lipoprotein-cholesterol; HDL-C, high density lipoprotein-cholesterol; LXRα, liver X receptor alpha; ABCG1/A1, ATP-binding cassette transporter G1/A1; apoE, apolipoprotein E; ICR, imprinting control region; GPx, glutathione peroxidase; IRS2, insulin receptor substrates 2; IDE, insulin-degrading enzyme; CDK, cyclin-dependent kinase; CUMS, chronic unpredictable mild stress; BDNF, brain derived neurotrophic factor; NGF, nerve growth factor; EnNSCs, endogenous neural stem cells; HMGB3, High mobility group protein B3; RBBP7, RB binding protein 7; EdU, 5-ethynyl-20-deoxyuridine; PCNA, proliferating cell nuclear antigen; NT3, neurotrophin-3; NeuN, neuronal nuclear protein; TGM, transglutaminase; FLG, filaggrin; HAS, hyaluronic acid synthases; IκBα, inhibitor of NF-kB; DNCB, 1-chloro-2,4-dinitrobenzene; TEWL, transepidermal water loss; SPINK5, serine protease inhibitor Kazal type-5; KLK, kallikrein; PAR2, protease activated receptor 2; REG3A, regenerating islet-derived protein 3-alpha; UUO, unilateral ureteral obstruction; MCP-1, monocyte chemoattractant protein-1; NX, nephrectomy; I/R, ischaemia reperfusion; MICP, mechanically induced constant pressure; LPS, lipopolysaccharide; BMDCs, bone marrow-derived dendritic cells; OVA, ovalbumin.

The bioactivity of CK has been described by inhibiting viability and proliferation, inducing apoptosis, by blocking the PI3K/mTOR/p70S6K1 signaling pathway (Table 4) [90]. Recently, many studies have been published comparing the health-promoting activities of CK and its derivatives (Table 4). Yang et al. identified the liposomal mediated improved anti-cancer activity of CK. They found that using GCKT-liposomes, the cellular uptake of GCK by lung adenocarcinoma (A549) cell line increased by enhancing time treatments (1, 2, and 4 h). The anti-tumor efficacy of GCKT-liposomes was observed to be more compared to CK. The use of GCKT-liposomes was advocated to overcome problems such as insufficient drug packaging, instable nature, rapid drug discharge, and poor blood circulation of conventional liposomes [33].

Similarly, CK-M described earlier in this review, were used for in vitro (A549 and PC-9) and in vivo models. The apoptosis percentage of CK-M was higher than free CK. Meanwhile, CK-M also displayed an improved tumor inhibitory effect in vivo [36]. The anti-lung cancer effects (in vitro and in vivo) of CK AP/TPGS mixed micelles [37] and CK PC/DP micellar system [38] have also been documented. Moreover, using in vitro models, BSA-CK NPs showed more significant cytotoxic effects in the liver carcinoma (HepG2), skin cell line (HaCaT), A549 cells, and colon cancer cell line (HT29) compared to monomer CK. However, for in vivo application, authors advocated the use of human serum albumin as an alternative to BSA to evade plausible immunologic concerns in humans [39]. In addition, CK-loaded GNPs have been identified as effectual photothermal and chemotherapeutic agents [40]. On a similar line, a higher dose-dependent inhibitory effect of chitosan nanoparticles loaded with CK (CK-NPs) was observed compared to CK. Authors described a lower (IC_50_ = 16.58 μg/mL) value for CK-NPs compared to CK (IC_50_ = 23.33 μg/mL) in HepG2 cells [41]. Likewise, CK loaded O-OCMC nanoparticles showed inhibitory effects against prostate cancer (PC3) cells through enhanced cytotoxicity and uptake of CK [42]. Chitosan polymer micelles decorated using A54 peptide, known as APD-CK, were utilized against Huh-7 and HepG2 cells. APD-CK showed higher cytotoxicity compared to CK and promoted apoptosis of HepG2 cells (Table 4) [43]. In another study, tLyp-1 (tumor-homing peptide) decorated liposomes loaded with parthenolide (active anti-tumor agent isolated from *Tanacetum parthenium*) and CK have been evaluated as lung cancer-targeting system. Enhanced anti-tumor activity was observed against both the cell line and animal model with limited adverse effects. In A549 cells, CK/parthenolide tLyp-1 liposomes decreased mitochondrial membrane potential and allowed greater Ca^2+^ efflux as well as significant inhibition of cell migration. From in vivo study, it was found that the complex has greater anti-tumor activity than the combination of these substances [91].

In another study, a derivative of CK, known as CKGal (20-O-β-D-lactopyranosyl-20(S)-protopanaxadiol) have been produced using β-1,4-galactosyltransferase, a regiospecific enzyme. The anti-proliferative action of CKGal was evaluated on skin melanoma (B16F10), brain carcinoma (U87MG), gastric cancer cell lines (AGS), and cervical carcinoma (HeLa). The CKGal displayed the best cytotoxicity against skin melanoma and cervical carcinoma compared to CK, Rh2, and F12 [92]. 

### 4.6. Neuroprotection

Several studies have documented the therapeutic effects of ginseng and ginsenosides in many central nervous system (CNS) ailments, for instance, Alzheimer’s disease, Parkinson’s disease, depression, and other ailments [6]. The protection could be ascribed to reducing neuroinflammation, neuroprotection, and regulating neurotransmitter release. In previous articles, the cognitive and neuroprotective role of CK has been described [3,11]. The current review summarizes the recent progress in neuroprotection effects of CK (Table 4). 

In a study, CK (derived from red ginseng) was able to exert neuroprotective effects in memory-impaired mouse (scopolamine-induced) model by inducing nuclear factor (erythroid-derived 2)-like 2 (Nrf2)-facilitated antioxidants. No effect was observed on the acetylcholine esterase (AChE) activity. In addition to that, CK defense against glutamate-induced cytotoxicity was also observed in HT-22 cells [93]. Recently, CK was found to be protective against memory and cognitive impairment in db/db mice by suppressing inflammation and oxidative stress, ameliorating dyslipidemia, insulin sensitivity, glucose tolerance as well as modulating ER stress and NLRP3 inflammasome pathway (Table 4) [94]. Moreover, in another study, CK minimized the morphine dependency by decreasing N-methyl-D-aspartate acid receptor subunit 1 activation in cultured cortical neurons from mice and frontal cortex of rat brains [95]. Another recent research documented the protective effects of CK against epilepsy in status epilepticus (SE) rat model. Compound K stimulated the release of gamma amino-butyric acid (GABA) and enhanced the GABA type A receptor subunit alpha1-facilitated inhibitory synaptic transmission [96]. In addition to the anti-atherosclerosis effects of CK and its derivatives as described above [68], the role of LXRα in immunomodulation has also been described in continuous research. Researchers demonstrated that CK was able to mitigate post-operative cognitive dysfunction (POCD). They found that CK inhibited hippocampal inflammation by activating LXRα. CK attenuated memory dysfunction by modulating Morris water-maze (MWM) test scores in an aged mouse model (Table 4) [97]. 

Regulation of aggregated amyloid-β (Aβ) is an important part of Alzheimer’s disease (AD) treatment. Concerning this, CK was found to enhance memory function, reduce Aβ expression and aggregation, and neuronal apoptosis by activating the Nrf2/Kelch-like ECH-associated protein-1 signaling pathway in ICR mice with diminished memory. Furthermore, the defensive effects were also due to the activation of the antioxidant system [98]. Likewise, the influence of the CK was elucidated using Aβ peptide-induced HT22 cells by improving viability, growth, and apoptosis of HT22 cells, as well as localization and expression of Aβ peptide in cells. Besides, ATP levels of cells were enhanced by increasing the activity of glucose transporters. Compound K restored abnormal energy metabolism (Aβ induced) by modulating the expressions of several enzymes (Table 4) [99]. Zong et al. first found that the CK mitigates cerebral discrepancies and Aβ_1-42_ accumulation in the hippocampus of chronic cerebral hypoperfusion-induced vascular dementia SD rats by enhancing the pSer9Glycogen synthase kinase-3b expression. To add further, CK upregulated PI3K/Akt pathway, resulting in increased insulin-degrading enzyme activity, a central enzyme accountable for degrading Aβ_1-42_ in the brain (Table 4) [100]. 

In an in vivo research, CK pretreatment resulted in the reduction of neurobehavioral score, water in brain tissue, and the cerebral infarct volume ratio against cerebral ischemia/reperfusion (I/R) injury in Wistar rats. Compound K enhanced activities of antioxidant enzymes, decreased levels of IL-1β, TNF-α, and declined level of high mobility group box 1 protein. Generally, the protective effects of CK against cerebral I/R damage may be due to anti-inflammatory and antioxidant bioactivities [101]. The earlier reviews have described the neuroprotective role of CK [3]. However, its antidepressant effect has been described recently. Depression is a global societal health concern. Concerning the effect of CK on depression, a study used two models: behavioral despair model (mice) and the chronic unpredictable mild stress (CUMS, rats) model. The antidepressant role may be due to regulation of the concentration of monoamine neurotransmitters, enhanced antioxidant capacity, and increased expressions of neuronal growth factor and brain-derived neurotrophic factor in the CNS [102]. In a follow-up study, the defensive effect of CK on CUMS depression was evaluated by inhibiting oxidative stress, inflammatory cytokines and NLRP3 expression using a mouse model [103]. 

Amongst others, recent work on explaining the beneficial effects of CK on hippocampal neurogenesis has been published. In one study, CK was found to induce neurogenesis and decrease apoptosis in thrombin-induced endogenous neural stem cells (EnNSCs) and improve animal prognosis by stimulating LXRα activation [104]. In another study, CK treatment triggered the proliferation of fresh cells and substantially increased their differentiation in the hippocampus (dentate gyrus) by activating brain derived neurotrophic factor (BDNF) signaling. The higher dose of CK was found to be more effective in aged mice compared to young ones (Table 4) [105]. 

### 4.7. Anti-Aging/Skin Protection

It is well-known that the production and synthesis of hyaluronic acid (HA) decreases with age. A study by Lim et al. identified that CK led to increased production of HA by activating Src (tyrosine kinase)-dependent Akt and ERK [106]. Ultraviolet type B (UVB) radiation induces photo- aging due to collagen degradation (type I and III) and increased production of iNOS, COX-2, and MMPs. Compound K supplementation diminished production of COX-2 and MMP-1 in NIH3T3 cells treated with UVB, and expression of type I collagen was modulated. Compound K also displayed the ability to improve the skin’s moisture level. The study also found skin hydration effects of CK in HaCaT cells (Table 4) [107]. Compound K-rich fraction applied to UVB-treated cells was equally successful in the prevention of UVB-mediated aging [108]. Interestingly, the effectiveness against reducing mRNA expression of MMP-1 was more in a synergistic approach by CK and maclurin (a natural compound) compared to individual compounds [109]. Another study assessed the role of CK in improving skin barrier function by upregulating the expression of serine protease inhibitor Kazal type-5 in atopic dermatitis-like mice and UVB-irradiated mouse model [110]. In recent research, CK improved imiquimod-induced psoriasis by inhibiting regenerating islet-derived protein 3 (RegIII) gamma expression in the mouse model and IL-36γ-induced Reg3A expression in human keratinocytes (Table 4) [111]. These findings advocate that CK plays a key role in the defense and anti-aging effects. 

### 4.8. Others 

Recently, a patented study investigating the beneficial effects of CK on renal tubulointerstitial lesions in C57BL/6 mice with unilateral ureteral obstruction identified the kidney-protective effects of CK. The results showed reduced production of pro-inflammatory cytokines and the prevention of leukocytes infiltration and fibrosis in the kidney. The positive outcomes were showcased by inhibiting NF-κB-associated priming, and by modulating STAT3 signaling and NLRP3 inflammasome activation (Table 4) [112]. Likewise, another study showed the protecting effects of CK on accelerated and severe lupus nephritis by hindering activation of NLRP3 inflammasome [113]. Ren and coworkers evaluated the protecting effects of CK and its analogues against asthma. Compound K and its analogues displayed a significant impact by reducing IgE and airway resistance [114]. Compound K pretreatment was reported to protect against cardiac I/R by activating the PI3K-Akt signaling pathway, which is crucial for autophagy-triggered apoptosis (Table 4) [115].

### 4.9. Clinical Studies 

#### 4.9.1. Anti-Diabetic 

Clinical studies evaluated the bioactivities of hydrolyzed ginseng extract. Recently, a double-blind, randomized controlled trial assessed the anti-glycaemic effects of hydrolyzed ginseng extract (GINST15, rich in CK) on prediabetic participants for 12 months. GINST15 resulted in improved fasting and 1-hour postprandial glucose levels. No effect was observed on 2-hour postprandial glucose levels [116]. An earlier clinical study also confirmed these results in which fermented red ginseng extract supplementation decreased fasting glucose, postprandial glucose levels and improved insulin levels in type 2 diabetic subjects compared to placebo [117]. 

#### 4.9.2. Neuroprotection

Flanagan et al. have documented the effects of GINST15 on hypo-pituitary-adrenal (HPA) and antioxidant activity clinically. In this double-blind, placebo-controlled, counterbalanced within-group study design different doses (high dose-960 mg and low dose-160 mg) of CK were given for 2 weeks. They found that, in response to severe exercise, CK supplementation resulted in dose-dependent declines in circulating cortisol and augmented antioxidant activity. This study first provided insights into the impact of ginseng treatment on the reactions to the stress associated with work [118]. A recent continued study by the same research group demonstrated direct evidence on task-related brain activity by evaluating CK’s treatment on behavioral performance and electroencephalography measures of cortical activity. After exercise, the upper and lower body response times were improved. Compound K augmented activity in cortical regions accountable for sustained attention, whereas exercise-triggered increases in arousal were diminished. In short, CK has been found to have inducible effects on the activity of the brain [119]. 

#### 4.9.3. Liver Protection

As mentioned above, in an in vivo study, GBCK25 has been found to have defensive effects against NASH [55]. On a similar line, recent research evaluated the protective effects of GBCK25 on liver function in a 12-week, randomized, double-blind clinical trial. The supplementation includes GBCK25 tablets (high (500) and low (125) mg/day) and placebo. Treatment at a low dose significantly reduced gamma-glutamyl transferase and high-sensitivity C-reactive protein levels in male participants, while high dose abridged fatigue score significantly. No side effects were observed for the supplementation. The study indicated that GBCK25 is safe and can improve liver function [120]. 

## 5. Concluding Remarks 

The review aimed at providing current information concerning the pharmacokinetics, safety, and health-promoting activities of CK and its derivatives for preventing and managing diseases. It is well known that the CK is more bioavailable than its parent saponins and has several health benefits. Although more bioavailable than major ginsenosides, CK has certain drawbacks that restrict its clinical use. The use of CK derivatives as nanocarriers were shown to have better permeability, solubility, and efflux, including enhanced health-promoting activities. The review provides new insight into CK derivatives to increase the metabolic proficiency of CK. Most health-promoting activities were in vitro and in vivo, including hepatoprotective, anti-inflammatory, anti-atherosclerosis, anti-cancer, neuroprotection, skin protection, and anti-aging. Besides, the limited number of clinical studies have also been documented. The diverse bioactivities of CK were based on modulating complicated signaling pathways and targeting various molecules. Compound K has been found to attenuate the activities of AMPK, MAPK, NF-kB, PI3K/Akt, mTOR/AMPK, JNK PI3K/mTOR/p70S6K1 signaling pathways. Overall, pharmacokinetic studies on monomer CK and its preclinical and clinical safety information data are limited. Further investigations are warranted to appraise the efficacy and safety of CK and its derivatives, especially in clinical studies.
